# A fast and versatile cross-linking strategy via *o*-phthalaldehyde condensation for mechanically strengthened and functional hydrogels

**DOI:** 10.1093/nsr/nwaa128

**Published:** 2020-06-12

**Authors:** Zhen Zhang, Chaoliang He, Yan Rong, Hui Ren, Tianran Wang, Zheng Zou, Xuesi Chen

**Affiliations:** CAS Key Laboratory of Polymer Ecomaterials, Changchun Institute of Applied Chemistry, Chinese Academy of Sciences, Changchun 130022, China; University of Science and Technology of China, Hefei 230026, China; CAS Key Laboratory of Polymer Ecomaterials, Changchun Institute of Applied Chemistry, Chinese Academy of Sciences, Changchun 130022, China; University of Science and Technology of China, Hefei 230026, China; CAS Key Laboratory of Polymer Ecomaterials, Changchun Institute of Applied Chemistry, Chinese Academy of Sciences, Changchun 130022, China; University of Chinese Academy of Sciences, Beijing 100049, China; CAS Key Laboratory of Polymer Ecomaterials, Changchun Institute of Applied Chemistry, Chinese Academy of Sciences, Changchun 130022, China; University of Science and Technology of China, Hefei 230026, China; CAS Key Laboratory of Polymer Ecomaterials, Changchun Institute of Applied Chemistry, Chinese Academy of Sciences, Changchun 130022, China; University of Science and Technology of China, Hefei 230026, China; CAS Key Laboratory of Polymer Ecomaterials, Changchun Institute of Applied Chemistry, Chinese Academy of Sciences, Changchun 130022, China; University of Science and Technology of China, Hefei 230026, China; CAS Key Laboratory of Polymer Ecomaterials, Changchun Institute of Applied Chemistry, Chinese Academy of Sciences, Changchun 130022, China; University of Science and Technology of China, Hefei 230026, China

**Keywords:** superfast gelation, *o*-phthalaldehyde chemistry, versatile cross-linking, functional hydrogel, reaction kinetics

## Abstract

Fast and catalyst-free cross-linking strategy is of great significance for construction of covalently cross-linked hydrogels. Here, we report the condensation reaction between *o*-phthalaldehyde (OPA) and *N*-nucleophiles (primary amine, hydrazide and aminooxy) for hydrogel formation for the first time. When four-arm poly(ethylene glycol) (4aPEG) capped with OPA was mixed with various *N*-nucleophile-terminated 4aPEG as building blocks, hydrogels were formed with superfast gelation rate, higher mechanical strength and markedly lower critical gelation concentrations, compared to benzaldehyde-based counterparts. Small molecule model reactions indicate the key to these cross-links is the fast formation of heterocycle phthalimidine product or isoindole (bis)hemiaminal intermediates, depending on the *N*-nucleophiles. The second-order rate constant for the formation of phthalimidine linkage (4.3 M^−1^ s^−1^) is over 3000 times and 200 times higher than those for acylhydrazone and oxime formation from benzaldehyde, respectively, and comparable to many cycloaddition click reactions. Based on the versatile OPA chemistry, various hydrogels can be readily prepared from naturally derived polysaccharides, proteins or synthetic polymers without complicated chemical modification. Moreover, biofunctionality is facilely imparted to the hydrogels by introducing amine-bearing peptides via the reaction between OPA and amino group.

## Teaser

A superfast and catalyst-free cross-linking strategy is innovatively developed based on the *o*-phthalaldehyde/*N*-nucleophiles condensation reaction, for facile construction of hydrogels with enhanced mechanical properties, superior biocompatibility and functional versatility.

## INTRODUCTION

Hydrogels are a class of viscoelastic materials that are 70%–99% water by mass, retained in a cross-linked polymer network. The high water content and viscoelastic nature of hydrogels render them physically similar to native tissues. These features provide the capacity to readily encapsulate cells and bioactive compounds, thus making hydrogels a particularly promising candidate for drug delivery and tissue engineering [1–4].

In hydrogels, hydrophilic or amphiphilic polymers are cross-linked to form a network by covalent bonds or physical interactions [5–9]. To date, a wide range of reactions have been exploited for the preparation of covalently cross-linked hydrogels, such as Michael additions, azide-alkyne cycloadditions and Diels-Alder reactions [10–14]. The condensation reactions between carbonyl groups and *N*-nucleophiles such as primary amine, hydrazide and aminooxy are widely used in the field of bioconjugation [15], as well as in the construction of hydrogels [16–19]. In particular, the formations of hydrazone and oxime can be considered click reactions due to their high reactivity, superb specificity and mild reaction conditions. However, compared with many cycloaddition-based reactions, the formations of hydrazone and oxime linkages proceed at much slower rates at neutral pH, with second-order rate constants that are commonly below 0.1 M^−1^ s^−1^ [20–22]. Although the gelation rates can be accelerated by tuning the pH to ca. 4.5 or by addition of aniline catalyst, these methods will compromise the biocompatibility of the materials and limit their biomedical applications [23]. Recently, Gillingham *et al*. reported that *o*-phthalaldehyde (OPA) is an exceptionally fast reactant with aminooxy in oxime condensations [24]. It was found that the reaction results in the rapid and irreversible formation of an isoindole bis(hemiaminal) (IBHA) heterocyclic intermediate, which gradually undergoes dehydration to yield oxime. Additionally, the reaction between OPA and primary amines has been shown to be a rapid and chemoselective condensation reaction for the formation of phthalimidine linkages under physiological conditions [25,26]. However, to date, there is no report on the feasibility of using the OPA/*N*-nucleophile condensation reaction as a potential cross-linking strategy for the construction of hydrogels with superior gelation kinetics and mechanical properties.

Here, we report a new method for the exceptionally fast formation of hydrogels via the reaction between OPA and *N*-nucleophiles. The formation of hydrogels was first demonstrated by using two types of four-arm poly(ethylene glycol) (4aPEG) as building blocks, one capped with OPA and the other with *N*-nucleophiles. Small molecule model reactions were conducted to illuminate the fast cross-linking mechanism and reaction kinetics. Moreover, the highly efficient and versatile OPA chemistry was exploited to construct diverse hydrogels from naturally derived polysaccharides, proteins or synthetic polymers, as well as to incorporate bioactive peptides into the hydrogels.

## RESULTS AND DISCUSSION

**Figure 1.**
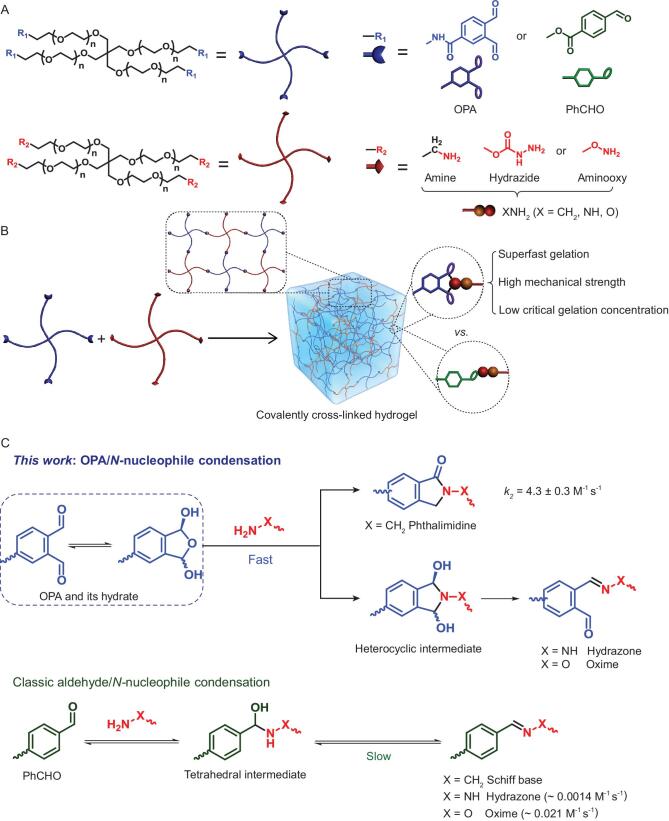
(A) Structures of building blocks for hydrogel formation: 4aPEG end-capped with OPA or *N*-nucleophiles (amine, hydrazide or aminooxy). Benzaldehyde (PhCHO) terminated 4aPEG was used for comparison. (B) Schematic representation of formation of hydrogel from materials in A. (C) Putative mechanism of the linkage formation between OPA (or PhCHO) and various *N*-nucleophiles with the molar ratio of 1:1. The new cross-linking strategy presented here is outlined in blue: OPA reacts with *N*-nucleophiles to give heterocyclic products, which greatly accelerate the initial bimolecular reaction.

**Figure 2.**
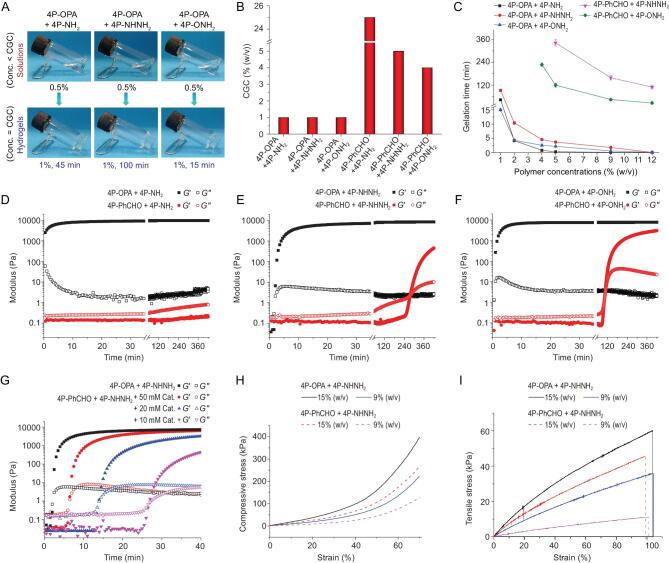
(A) Photographs showing the solution or hydrogel status of the mixtures of 4P-OPA with 4P-NH_2_, 4P-NHNH_2_ and 4P-ONH_2_, respectively, when the polymer concentrations were below or equal to the critical gelation concentrations (CGCs). (B) CGCs for various combinations of 4aPEG. (C) Gelation time at different polymer concentrations determined by vial inversion method (mean ± SD, n = 3). (D–F) Time sweep rheological measurement for the 5% (w/v) mixtures of various formulations. (G) Time sweep rheological measurement for the 5% (w/v) mixtures of 4P-OPA with 4P-NHNH_2_, compared with those for the 5% (w/v) mixtures of 4P-PhCHO with 4P-NHNH_2_ in the presence of 10–50 mM of aniline catalyst. (H) Representative stress-strain curves for compressive tests. (I) Representative stress-strain curves for tensile tests. The molar ratios of reactive end groups of all the mixtures in (A-I) were fixed at 1:1.

To obtain the building blocks for the construction of hydrogels via OPA/*N*-nucleophile condensation, multiple forms of 4aPEG (*M_n_* = 10 kDa) end-capped with OPA or *N*-nucleophiles were synthesized, including OPA-terminated 4aPEG (4P-OPA), primary amine-terminated 4aPEG (4P-NH_2_), hydrazide-terminated 4aPEG (4P-NHNH_2_) and aminooxy-terminated 4aPEG (4P-ONH_2_). Benzaldehyde-terminated 4aPEG (4P-PhCHO), a routinely employed building block, was also prepared for comparison (Fig. 1; supporting information: Scheme S1, Figs S1–S11). With the molar ratio of reactive end groups fixed at 1:1, the gelation behaviors for various mixtures of 4aPEG were estimated by the vial inversion method at pH 7.4 and 37°C. It was observed that all the 4P-OPA-based mixtures displayed CGCs as low as 1% (w/v), which is defined as the lowest total polymer concentration required for hydrogel formation within 24 h (Fig. 2A and B). Free-standing hydrogels were formed within 10 min after mixing 4P-OPA with 4P-NH_2_, 4P-NHNH_2_ or 4P-ONH_2_, even at a low polymer concentration of 2% (w/v) (Fig. 2C). The gelation of the 4P-OPA-based systems was accelerated further with increasing polymer concentration, and hydrogels formed instantly after mixing the precursor components when the polymer concentration was higher than 9% (w/v).

By contrast, after mixing 4P-PhCHO with 4P-NH_2_, hydrogel could only form at polymer concentration not less than 25% (w/v) (Fig. 2B, Fig. S12). Although hydrogels formed after mixing 4P-PhCHO with 4P-NHNH_2_ or 4P-ONH_2_ at relatively low polymer concentrations (CGCs = 5% (w/v) and 4% (w/v), respectively), the gelation time was rather long: 110–341 min was required for the gelation of 4P-PhCHO with 4P-NHNH_2_ and 27–227 min for 4P-PhCHO with 4P-ONH_2_, depending on the polymer concentration (Fig. 2C).

Rates of hydrogel formation were studied further by rheometry, using time sweep measurement. The storage modulus (*G′*) and loss modulus (*G″*) were recorded immediately after mixing the precursor polymers. The time at the crossover of *G′* and *G″* was regarded as the gelation point. As shown in Fig. 2D–F, for the 5% (w/v) 4P-OPA-based systems, the *G′* increased rapidly within the first 5 min, indicating fast hydrogel formation. Meanwhile, there was no obvious *G′* increase within 90 min for all the 4P-PhCHO based counterparts. These results are in agreement with the above results from vial inversion. Specifically, it was found that a hydrogel status with *G'* > *G″* was formed before the first data point was recorded for the 5% (w/v) mixture of 4P-OPA with 4P-NH_2_, suggesting an instantaneous gelation process (Fig. 2D). In sharp contrast, the combination of 4P-PhCHO with 4P-NH_2_ at 5% (w/v) only resulted in a liquid mixture with *G′* < *G″* throughout the measurement. Additionally, the gelation point for the 5% (w/v) mixture of 4P-OPA with 4P-NHNH_2_ was determined as 2 min through the *G′*/*G″* crossover method, while it was prolonged to 277 min by mixing 4P-PhCHO with 4P-NHNH_2_ (Fig. 2E).

Apart from their exceptionally fast gelation kinetics, significantly higher *G′* values were obtained for the 4P-OPA-based hydrogels, indicating enhanced mechanical strength. For instance, the *G′* of 5% (w/v) 4P-OPA-based hydrogels rapidly increased to 7000–9200 Pa within 30 min and maintained at 7700–9700 Pa at 420 min, compared to the much lower *G′* for the counterparts of 4P-PhCHO with 4P-NHNH_2_ or 4P-ONH_2_ (450 and 3100 Pa at 420 min, respectively) (Fig. 2D–F). Frequency sweep tests showed that the *G′* of 5% (w/v) 4P-OPA-based hydrogels were nearly constant from 0.1 Hz to 100 Hz. Strain sweep tests revealed that the hydrogels exhibited linear viscoelastic behavior at strain from 0.1% to 20%, while the *G′* rapidly decreased at large strain (Fig. S13). In addition, the *G′* of the 4P-OPA-based hydrogels can be well tuned from 340 Pa to over 19 000 Pa by changing the polymer concentration from 2% (w/v) to 9% (w/v) (Fig. S14).

Nucleophilic catalysts have been developed to accelerate the formation of hydrazone and oxime linkages by virtue of the first formation of Schiff bases [27]. Thus, to investigate if the 4P-OPA-based hydrogels still hold the advantage in gelation kinetics, time sweep rheological measurement of 4P-PhCHO based hydrogels was performed with the presence of 10–50 mM of classic aniline catalyst (Fig. 2G). For the 5% (w/v) mixtures of 4P-PhCHO with 4P-NHNH_2_, the addition of 10 mM aniline dramatically shortened the gelation point to 27 min. Increasing the catalyst concentration to 20 and 50 mM further reduced the gelation point to 14 and 6.5 min, respectively. Even though the addition of aniline catalyst significantly accelerated the gelation of 4P-PhCHO based system, the mixture of 4P-OPA with 4P-NHNH_2_ (5% (w/v)) still exhibited an obviously faster gelation rate. Similar results were obtained for the mixture of 4P-OPA with 4P-ONH_2_, which exhibited superior gelation ability as compared with its 4P-PhCHO counterpart with the presence of aniline catalyst (Fig. S15). Additional gelation kinetics were conducted to investigate the effects of pH. As the pH value increased from 6.2 to 8.0, the gelation rate of 4P-OPA-based hydrogels increased unexpectedly (Fig. S16). This behavior is opposite to what was observed for the 4P-PhCHO based systems, for which gelation proceeded faster at acidic pH.

Moreover, compressive and tensile tests were performed to evaluate the mechanical strength of bulk hydrogels. As expected, the compressive and tensile strength of the 4P-OPA/4P-NHNH_2_ hydrogels increased from 234 kPa and 37.7 kPa to 410 kPa and 60.5 kPa, respectively, with increasing the polymer concentration from 9% (w/v) to 15% (w/v) (Fig. 2H and I, Fig. S17). At the same polymer concentrations, the 4P-OPA/4P-NHNH_2_ hydrogels exhibited significantly strengthened mechanical properties as compared with the 4P-PhCHO/4P-NHNH_2_ counterparts. Scanning electron microscopy revealed a porous structure of the freeze-dried 4P-OPA/4P-NHNH_2_ hydrogels (Fig. S18).

It is noteworthy that the condensation reactions between carbonyl groups and *N*-nucleophiles are currently widely used cross-linking approaches for hydrogel formation. Specifically, imine linkages are readily hydrolyzed in aqueous solutions, while hydrazone- and oxime-based linkages, much more stable than imines, form slowly at neutral pH. Mechanism studies by Jencks suggested that carbonyl condensations typically undergo a rapid pre-equilibrium formation of a tetrahedral hemiaminal intermediate, followed by an acid-catalyzed and rate-limiting dehydration (Fig. 1C) [28–30]. As a consequence, synthesis of 4P-PhCHO based hydrogels usually needs high polymer concentrations, slightly acidic media or nucleophilic catalysts, all of which are detrimental to biomedical applications [31–33]. In the case of 4P-OPA-based systems, the markedly increased gelation rate can be attributed to the increased reactivity of OPA to *N*-nucleophiles, resulting from the strong substituent effects of the two adjacent formyl groups [34]. More importantly, OPA provides an intramolecular trap such that more stable heterocycle products (phthalimidine for primary amine, and IBHA intermediate for hydrazide or aminooxy) are formed, which greatly accelerates the speed of initial bimolecular addition reaction [24].

**Figure 3.**
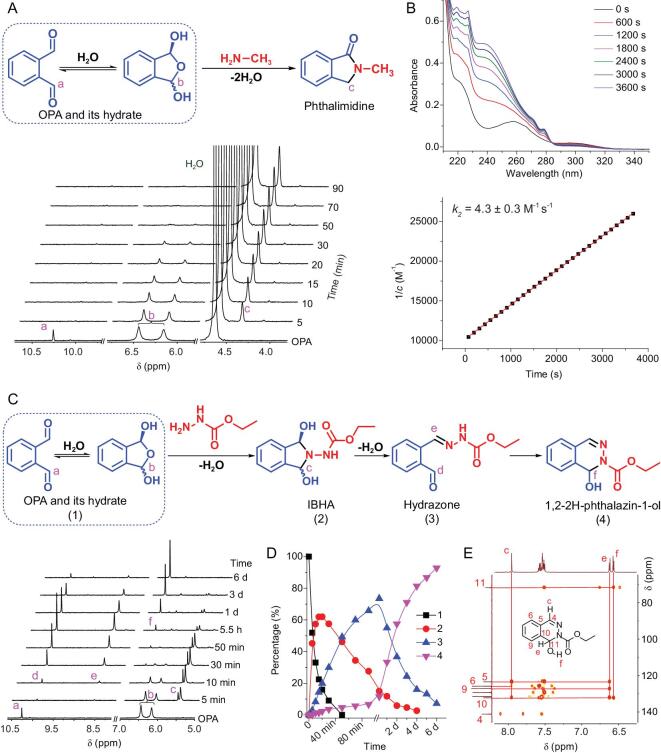
(A) Time-dependent ^1^H NMR analysis of the reaction of OPA with methylamine in D_2_O/DMSO-*d*_6_ (4:1 (v/v)). (B) Kinetic profile of phthalimidine formation monitored by UV-vis spectroscopy (mean ± SD, n = 3). (C) Time-dependent ^1^H NMR analysis of the reaction of OPA with ethyl carbazate in D_2_O/DMSO-*d*_6_ (4:1 (v/v)). (D) The percentages of compounds are calculated using ^1^H NMR integration. The numbers and the corresponding chemical structures in C are indicated. (E) ^1^H-^13^C HMBC spectrum of 2-(ethoxycarbonyl)-1,2-dihydro-phthalazin-1-ol in DMSO-*d*_6_.

To better understand the cross-linking mechanism of the 4P-OPA-based hydrogels, model reactions between commercially available OPA and *N*-nucleophiles (methylamine, ethyl carbazate and ethoxyamine) were investigated by nuclear magnetic resonance (NMR) spectrometry, UV-vis spectrometry and mass spectrometry. It has been established that OPA exists reversibly as a hydrate in aqueous solution [35]. From the ^1^H NMR spectrum of OPA in D_2_O/DMSO-*d*_6_ (4:1 (v/v)), the chemical shifts assigned to OPA hydrate were observed at 6.44 and 6.15 ppm (Fig. 3A, Fig. S19), corresponding to the acetal protons (*meso* form and diastereomer). At 5 min after mixing OPA with methylamine, the aldehyde peak of OPA at 10.26 ppm was almost completely eliminated, with the generation of a phthalimidine product (4.37 ppm). At the same time, the peak intensities for OPA hydrate kept decreasing, while these for phthalimidine tended to increase. The reaction nearly completed within 50 min, resulting in clean NMR and ESI-MS spectra corresponding to single phthalimidine product (Fig. S20).

The formation kinetics of phthalimidine was further assessed via UV-vis spectrometry by mixing 0.1 mM of OPA with 0.1 mM of methylamine in phosphate buffer saline (PBS) (Fig. 3B, Fig. S21). The progress of reaction was monitored by recording the absorption at 238 nm over time. Fitting of kinetic plot of the reaction according to a second-order kinetic equation gave a rate constant of 4.3 M^−1^ s^−1^ for phthalimidine formation, which is over 3000 times and 200 times higher than those for acylhydrazone (0.00141 M^−1^ s^−1^) and oxime (0.0213 M^−1^ s^−1^) formation from benzaldehyde in phosphate buffer, respectively [36]. Moreover, the rate constant for phthalimidine formation is in the same level as those of many other click reactions, such as strain-promoted alkyne-azide cycloaddition (SPAAC) (1 M^−1^ s^−1^) and inverse electron demand Diels-Alder (iEDDA) reaction (1–10 M^−1^ s^−1^) [21]. Thus, this result strongly supports the vast potential of OPA/*N*-nucleophile condensation as a useful tool for click chemistry.

With regard to the reaction of OPA with ethyl carbazate or ethoxyamine, time-dependent ^1^H NMR analysis suggested the formation of IBHA intermediate, which gradually dehydrated to form the hydrazone or oxime. For instance, after mixing OPA with ethyl carbazate for 5 min, ^1^H NMR spectrum showed that the mixture was composed of OPA hydrate and IHBA (5.67 and 5.45 ppm) with an OPA hydrate/IHBA molar ratio of 53:47 (Fig. 3C and D). Then, the peak intensities for OPA hydrate kept decreasing, with the continuous generation of the expected hydrazone (Figs S22 and S23). Due to the fact that the residual aldehyde group was able to engage in another nucleophilic attack, the hydrazone formed by OPA with ethyl carbazate could undergo an intramolecular cyclization within several days to yield a cyclization pro-duct, 2-(ethoxycarbonyl)-1,2-dihydro-phthalazin-1-ol (Fig. 3E, Figs S24 and S25).

Additionally, it is worth noting that the hydrazone could also react with the second ethyl carbazate to form a bis-hydrazone precipitate with one OPA linked to two hydrazides, especially when excessive ethyl carbazate was added (Fig. S26). On the other hand, oxime product was formed from the reaction between OPA and ethoxyamine (Figs S27 and S28).

**Figure 4.**
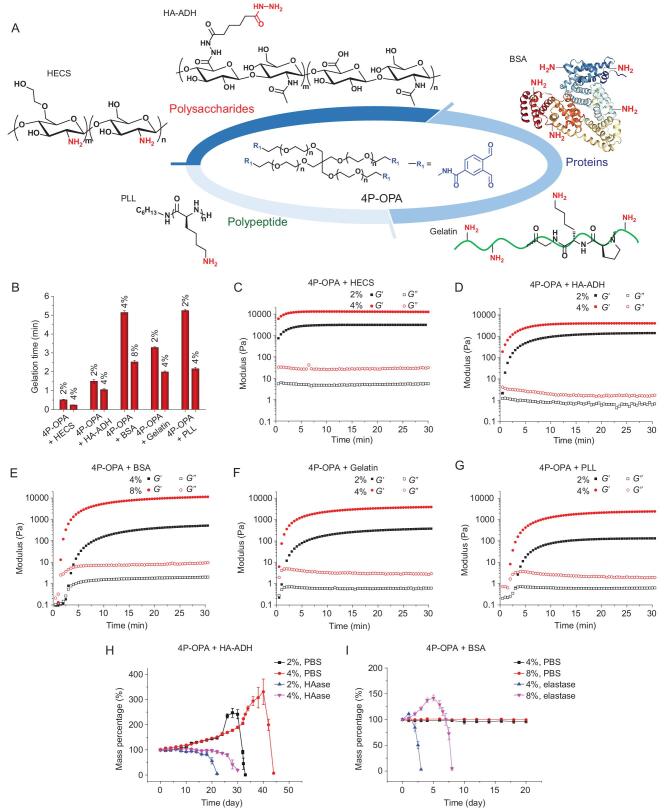
(A) Schematic illustration for the reactions between 4P-OPA and various *N*-nucleophile-containing naturally derived or synthetic macromolecules, including hydroxyethyl chitosan (HECS), adipic dihydrazide modified hyaluronic acid (HA-ADH), bovine serum albumin (BSA), gelatin and poly(L-lysine) (PLL). (B) Gelation time obtained by vial inversion test for the mixture of 4P-OPA with HECS, HA-ADH, BSA, gelatin or PLL at different polymer concentrations (mean ± SD, n = 3). (C–G) Time sweep rheological measurement of the gelation process for each combination in A. Swelling and degradation profiles of (H) 4P-OPA/HA-ADH hydrogels and (I) 4P-OPA/BSA hydrogels. The concentration of hyaluronidase (HAase) and elastase is 10 U mL^−1^ and 1 U mL^−1^, respectively (mean ± SD, n = 3). 4P-OPA and each of *N*-nucleophile-containing macromolecules were mixed at a mass ratio of 1:1.

The high reactivity of OPA toward *N*-nucleophiles provides a versatile strategy for fabrication of diverse hydrogels from both naturally derived and synthetic macromolecules. To verify the feasibility of this approach, we further investigated the gelation properties of 4P-OPA with various *N*-nucleophile-containing naturally derived and synthetic macromolecules, including hydroxyethyl chitosan (HECS), adipic dihydrazide modified hyaluronic acid (HA-ADH), gelatin, bovine serum albumin (BSA) and poly(L-lysine) (PLL) (Fig. 4A). All combinations were mixed at a mass ratio of 1:1 in PBS. Vial inversion tests and time sweep rheological measurements showed that hydrogels could be formed within 15 s to 5 min after mixing 4P-OPA with each of these macromolecules, depending on the type of *N*-nucleophile-containing macromolecule and polymer concentration (Fig. 4B). Increasing the polymer concentration would decrease the gelation time and markedly increase the mechanical strength of resultant hydrogels (Fig. 4C–G). The incorporation of diverse naturally derived or synthetic macromolecules endows the hydrogels with adjustable biodegradability. For instance, the 4P-OPA/HA-ADH hydrogels could be degraded within 22 days and 30 days at the polymer concentrations of 2% (w/v) and 4% (w/v), respectively, in the presence of 10 U mL^−1^ of hyaluronidase (Fig. 4H). The 4P-OPA/BSA hydrogels could be rapidly degraded within 3 days and 8 days at the polymer concentration of 4% (w/v) and 8% (w/v), respectively, in the presence of 1 U mL^−1^ of elastase (Fig. 4I). Besides, gelatin and hyaluronic acid are known to promote cell adhesion and proliferation [37,38], and chitosan possesses hemostatic and anti-bacterial activity [39]. Therefore, the results indicated that 4P-OPA is suitable to serve as a universal building block for construction of hydrogels to satisfy different requirements for biomedical applications.

**Figure 5.**
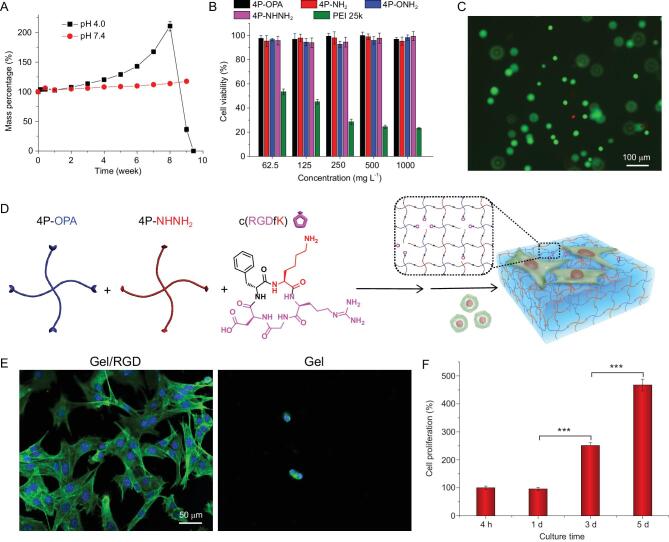
(A) Swelling and degradation profiles of the 5% (w/v) 4P-OPA/4P-NHNH_2_ hydrogels in PBS at pH 4.0 and 7.4, respectively (mean ± SD, n = 3). (B) Viability of L929 cells after incubation with end-group modified 4aPEG for 48 h determined by MTT assay (mean ± SD, n = 6). A widely used cationic polymer, polyethyleneimine (PEI) 25k, was used as a positive control. (C) Live/dead cell staining images of L929 cells encapsulated within 2% (w/v) 4P-OPA/4P-NHNH_2_ hydrogels for 24 h (green: live; red: dead). (D) Schematic illustration for functionalization of 4P-OPA/4P-NHNH_2_ hydrogels with amine-bearing bioactive peptide through the reaction between OPA and amino group. (E) Confocal laser scanning microscope (CLSM) images for the NIH 3T3 cells on the hydrogel surface with or without RGD modification after seeding for 12 h. The nuclei and F-actin filaments were stained with DAPI and Alexa Fluor 488 phalloidin, respectively. (F) Proliferation of NIH 3T3 cells on RGD modified 4aPEG hydrogels determined by CCK-8 method (mean ± SD, ***P < 0.001, One-Way ANOVA, n = 3).

Subsequently, the swelling and degradation behaviors of the 4P-OPA-based hydrogels in PBS were evaluated at pH 7.4 (physiological pH) and 4.0 (acidic conditions that can catalyze the hydrolysis of carbon-nitrogen double bonds). The 5% (w/v) 4P-OPA/4P-NHNH_2_ hydrogel exhibited good hydrolytic stability for nine weeks at pH 7.4 (Fig. 5A). In contrast, the same hydrogels showed an obvious increase in the swelling ratio at pH 4.0 in the first eight weeks, likely associated with the gradual decline in the cross-linking density caused by the continuous hydrolysis of the hydrazone-containing linkages under acidic conditions [40]. After incubation for nine weeks at pH 4.0 the hydrogels were found to have dissolved in the buffer, suggesting the complete degradation of the hydrogel. Additionally, the 4P-OPA/4P-NH_2_ and 4P-OPA/4P-ONH_2_ hydrogels showed no obvious degradation after nine weeks at both pH 7.4 and 4.0 (Fig. S29). These results indicated that the phthalimidine and oxime linkages formed by the 4P-OPA/4P-NH_2_ and 4P-OPA/4P-ONH_2_ systems, respectively, possess high hydrolytic stability [24–26]. The slow degradation profiles of 4P-OPA-based hydrogels offer the potential of long-term application of these materials *in vivo* at a relative low polymer concentration, given that hydrogels with high polymer content may induce severe inflammation or toxic reactions [41].

Excellent biocompatibility of both the precursor polymers and the gelation process is required if hydrogels formed *in situ* are intended for biomedical applications. The cytotoxicity of the end-group modified 4aPEG macromonomers was evaluated by MTT assay against L929 fibroblasts, with polyethyleneimine (PEI) 25k as a positive control. As shown in Fig. 5B, the cell viability remained over 90% when cultured with the 4aPEG-based macromonomers at all concentrations up to 1000 mg L^−1^ for 48 h, indicating no detectable cytotoxicity of the hydrogel precursor polymers. To further evaluate the cytocompatibility of the gelation process of the 4P-OPA-based hydrogels, *in situ* encapsulation of L929 cells by the 4P-OPA/4P-NHNH_2_ hydrogels was carried out. After 24 h of incubation, the viability of the cells cultured inside the hydrogels was evaluated by live/dead cell staining kit. As shown in Fig. 5C, the majority of the cells were stained green with calcein, suggesting high viability of cells inside the hydrogels and therefore good cytocompatibility of the gelation process.

Further, the chemoselective and traceless reaction between OPA and amino group offers a facile approach to introducing amino-containing functional molecules into hydrogel networks. To demonstrate this concept, 4P-OPA was mixed with 4P-NHNH_2_ and 2 mM of c(RGDfK), a cyclic cell adhesion peptide containing a lysine residue (Fig. 5D). The condensation reaction of OPA with the ϵ-amino group of the lysine residue led to the conjugation of c(RGDfK) into the hydrogel network. To investigate the variation in cell-adhesive property of the hydrogels after incorporation of c(RGDfK), the adhesion and spread morphology of NIH 3T3 fibroblasts were observed after seeding on the hydrogel surface for 12 h. The cell nuclei were stained with 4',6-diamidino-2-phenylindole (DAPI) and F-actin filaments were stained with Alexa Fluor 488 phalloidin. On the c(RGDfK) modified 4aPEG hydrogels, filopodia and lamellipodia were observed at the leading edge of fibroblasts with actin bundles (Fig. 5E), suggesting healthy cell adhesion and spread. Moreover, the cells were found to adhere on the hydrogel surface and proliferate over 5 days as manifested by the cell counting kit-8 (CCK-8) assay (Fig. 5F). By contrast, very few cells were found to adhere or spread on the 4aPEG hydrogels without c(RGDfK) modification. Thus, our results clearly demonstrated the potential of OPA chemistry as a straightforward method to incorporate bioactive peptides or proteins into hydrogels.

## CONCLUSION

Here we have presented an exceptionally fast gelation approach based on the reaction between OPA and *N*-nucleophiles (primary amine, hydrazide or aminooxy group). Compared to the counterparts formed by traditional benzaldehyde/*N*-nucleophile reactions, the OPA/*N*-nucleophile cross-linked 4aPEG hydrogels displayed markedly faster gelation rates, superior mechanical strengths and lower critical gelation concentrations under physiological conditions. The model reactions between OPA and small molecule *N*-nucleophiles revealed that the key to the cross-links is the rapid formation of heterocyclic phthalimidine linkage or isoindole (bis)hemiaminal intermediate. The rate constant determined by a second-order kinetic model between OPA and methylamine was 4.3 M^−1^ s^−1^, which is over 3000 times and 200 times higher than those for acylhydrazone and oxime formation from benzaldehyde, respectively, and in the same level as many widely used cycloaddition-based click reactions. Both the precursor polymers and the OPA-based cross-linking process exhibit good cytocompatibility. Additionally, the highly efficient and versatile reaction of OPA with *N*-nucleophiles allows easy conjugation of bioactive peptides into the hydrogel network, as well as facile preparation of diverse hydrogels from naturally derived polysaccharides, proteins and synthetic polymers. To our knowledge, this is the first study of utilization of OPA/*N*-nucleophile chemistry as the cross-linking approach for hydrogel formation. The fast gelation kinetics allow the facile encapsulation of cells in hydrogels for three-dimensional culture. Moreover, the efficient and traceless reaction of OPA with amino groups in proteins of soft tissues can be exploited to prepare hydrogel bioadhesives for wound closure. Considering the remarkable advantages of the OPA chemistry, we believe this rapid catalyst-free cross-linking strategy holds tremendous potential for the construction of hydrogels for a wide range of biomedical applications.

## Supplementary Material

nwaa128_Supplemental_File
