# Supplementary material for: A fast and versatile cross-linking strategy via o-phthalaldehyde condensation for mechanically strengthened and functional hydrogels
Source: Natl Sci Rev. 2020 Jun 12;8(4):nwaa128. doi: 10.1093/nsr/nwaa128 (PMC8288384; doi:10.1093/nsr/nwaa128)
Supplement: nwaa128_Supplemental_File [file nwaa128_supplemental_file.docx]

Supporting information for:

A fast and versatile cross-linking strategy via o-phthalaldehyde condensation for mechanically strengthened and functional hydrogels

Zhen Zhang^†‡^, Chaoliang He^†‡*^, Yan Rong^†#^, Hui Ren^†‡^, Tianran Wang^†‡^, Zheng Zou^†‡^, and Xuesi Chen^†‡*^

^†^CAS Key Laboratory of Polymer Ecomaterials, Changchun Institute of Applied Chemistry, Chinese Academy of Sciences, Changchun 130022, P. R. China

^‡^University of Science and Technology of China, Hefei 230026, P. R. China

^#^University of Chinese Academy of Sciences, Beijing 100049, P. R. China

Supplementary experimental sections:

1. Materials and methods

4-arm PEG (4aPEG, *M_n_* = 10 kDa as determined by MALDI-TOF) was purchased from Pharmicell Co., Inc. (Seoul, Republic of Korea). 4-Formyl benzoic acid, 1-(3-dimethylaminopropyl)-3-ethylcarbodiimide hydrochloride (EDC·HCl), 4-dimethylaminopyridine (DMAP), phthalimide, triphenylphosphine, diisopropyl azodicarboxylate (DIAD), *N*-hydroxyphthalimide, 4-nitrophenyl chloroformate (NPC), *N*-bromosuccinimide, benzoyl peroxide, scandium(III) trifluoromethanesulfonate (Sc(OTf)_3_), *N*-hydroxysuccinimide (NHS), and ethoxyamine hydrochloride were purchased from J&K Scientific Ltd. (Beijing, China). Hydrazine hydrate and DCl (20 wt% in D_2_O, 100.0 atom % D) were purchased from Acros Organics (Belgium). 3,4-Dimethylbenzoic acid, *o*-phthalaldehyde (OPA) (98%), methylamine hydrochloride, and ethyl carbazate (97%) were purchased from Alfa Aesar (Ward Hill, Massachusetts, USA). Hydroxyethyl chitosan (HECS, *M_n_* = 35 kDa, polydispersity (PDI) = 2.49) was purchased from Sigma-Aldrich (St. Louis, Missouri, USA). Hyaluronic acid (HA, *M_n_* = 73 kDa, PDI = 2.19) was bought from Bloomage Biotech Co., Ltd. (Jinan, Shandong, China). Adipic dihydrazide modified hyaluronic acid (HA-ADH) was synthesized according to a method described previously,^1^ and the *M_n_* and PDI of HA-ADH were determined by gel permeation chromatography (GPC) to be 56 kDa and 2.5, respectively. Poly(L-lysine) (PLL) was synthesized *via* the ring-opening polymerization of L-lysine-*N*-carboxyanhydride using n-hexylamine as the initiator according to our previous method,^2^ and the degree of polymerization (DP) of PLL was determined to be 200 by ^1^H NMR. Gelatin (from bovine skin, type B, gel strength ~225 bloom) was obtained from Solarbio Life Sciences (Beijing, China). Bovine serum albumin (BSA, 66 kDa) was purchased from Sigma-Aldrich (St. Louis, Missouri, USA). Other reagents were purchased from manufacturer and used without further purification.

NMR spectra were recorded on a Bruker Avance III NMR spectrometer (Bruker Daltonics Inc.) at 298 K. Chemical shifts are reported as *δ* in parts per million (ppm) and referenced to the chemical shift of the residual solvent resonances (DMSO: ^1^H *δ* = 2.50 ppm, CDCl_3_: ^1^H *δ* = 7.28 ppm). Rheological experiments were conducted on a rheometer (MCR 302, Anton Paar GmbH., Austria) with a flat upper plate (diameter 25 mm). The cross-section structure of the hydrogels in lyophilized state was observed on a scanning electron microscope (GeminiSEM, Carl Zeiss AG, Germany). The hydrogels were frozen in liquid nitrogen and fractured mechanically before freeze-drying. The pH was monitored by a HI2211 pH/ORP meter equipped with HI1131 glass electrode (Hanna Instruments Inc., Italy). Molecular weight distribution was determined by gel permeation chromatography (GPC) with *N*,*N*-dimethylformamide as eluent.

2. Synthesis

**2.1 Benzaldehyde terminated 4-arm PEG (4P-PhCHO)**

4P-PhCHO was synthesized by Steglich esterification between 4aPEG and 4-formyl benzoic acid with EDC·HCl and DMAP. Typically, dry 4aPEG (5 g, 2 mmol of OH), 4-formyl benzoic acid (0.9 g, 6 mmol, 3 eq), EDC·HCl (2.3 g, 12 mmol), and DMAP (146 mg, 1.2 mmol) were dissolved in anhydrous CH_2_Cl_2_ (50 mL). The mixture was stirred at r.t. for 3 days. The solvent was evaporated in vacuo and the residue was dissolved in deionized water. The crude product was dialyzed for 3 days and lyophilized as a white fluffy solid in nearly quantitative yield. ^1^H NMR (300 MHz, CDCl_3_, δ): 10.11 (s, 1H; CHO), 8.22 (m, 2H; Ar H), 7.96 (m, 2H; Ar H), 4.51 (m, 2H; CH_2_), 3.64 (s, 250H; PEG H). ^1^H NMR spectroscopy indicated 96% conversion of the end group to benzaldehyde.

**2.2 Primary amine terminated 4-arm PEG (4P-NH_2_)**

4P-NH_2_ was synthesized by Mitsunobu reaction between 4aPEG and phthalimide with DIAD and triphenylphosphine, followed by hydrazinolysis.^3^ Dry 4aPEG (5 g, 2 mmol of OH), phthalimide (1.47 g, 10 mmol, 5 eq) and triphenylphosphine (2.62 g, 10mmol, 5eq) were dissolved in anhydrous THF (40 mL). DIAD (2.03 g, 10 mmol, 5 eq) in THF (10 mL) was added dropwise. The mixture was stirred at r.t. for a week. After that, the solvent was removed in vacuo. The residue was dissolved in water and filtered. The aqueous solution was saturated with NaCl and extracted by CH_2_Cl_2_. The organic phase was dried over Na_2_SO_4_, filtered, and concentrated. The product was then precipitated from the organic phase thrice using diethyl ether and obtained as a pale yellow powder (3.5 g, 70%). ^1^H NMR (300 MHz, CDCl_3_, δ): 7.77 (m, 4H; Ar H), 3.63 (s, 259H; PEG H). ^1^H NMR spectroscopy indicated 93% conversion of the end group.

N_2_H_4_·H_2_O (2.3 mL, > 30 eq) was added to phthalimido-substituted 4aPEG in THF (50 mL). The solution was stirred at r.t. for 60 h, filtered, and evaporated in vacuo. The residue was dissolved in CH_2_Cl_2_ and precipitated in diethyl ether twice to yield 4P-NH_2_ as a pale yellow powder (2.9 g, 83%). For hydrogel formation, 4P-NH_2_ was dialyzed in deionized water for 2 days and lyophilized. ^1^H NMR (300 MHz, CDCl_3_, δ): 3.64 (s, 300H; PEG H), 2.94 (t, 2H; CH_2_). ^1^H NMR spectroscopy indicated 80% conversion of the end group. PDI = 1.37.

**2.3 Aminooxy terminated 4-arm PEG (4P-ONH_2_)**

4P-ONH_2_ was synthesized by Mitsunobu reaction between 4aPEG and *N*-hydroxyphthalimide with DIAD and triphenylphosphine, followed by hydrazinolysis. Dry 4aPEG (5 g, 2 mmol of OH), *N*-hydroxyphthalimide (1.47 g, 10 mmol, 5 eq) and triphenylphosphine (2.62 g, 10mmol, 5eq) were dissolved in anhydrous THF (40 mL). DIAD (2.03 g, 10 mmol, 5 eq) in THF (10 mL) was then added dropwise. The mixture was stirred at r.t. for 3 days. After that, the solvent was removed in vacuo. The residue was dissolved in water and filtered. The aqueous solution was washed with diethyl ether thrice, saturated with NaCl and extracted by CH_2_Cl_2_. The organic phase was dried over Na_2_SO_4_, filtered, and concentrated. The product was obtained from the organic phase by precipitation with diethyl ether as a white powder (3.8 g, 76%). ^1^H NMR (300 MHz, CDCl_3_, δ): 7.80 (m, 4H; Ar H), 4.38 (m, 2H; CH_2_), 3.65 (s, 258H; PEG H). ^1^H NMR spectroscopy indicated 93% conversion of the end group.

N_2_H_4_·H_2_O (2.3 mL, > 30 eq) was added to phthalimidooxy-substituted 4aPEG in THF (50 mL). The solution was stirred at r.t. for 60 h, filtered, and evaporated in vacuo. The residue was dissolved in deionized water, dialyzed for 2 days, and lyophilized in nearly quantitative yield. ^1^H NMR (300 MHz, CDCl_3_, δ): 3.65 (s; PEG H). PDI = 1.22.

**2.4 Hydrazide terminated 4-arm PEG (4P-NHNH_2_)**

Dry 4aPEG (5 g, 2 mmol of OH) was dissolved in anhydrous CH_2_Cl_2_ (30 mL), to which NPC (2.02 g, 10 mmol, 5 eq) in CH_2_Cl_2_ (20 mL) was added dropwise. The mixture was stirred at r.t. overnight, washed with brine, dried over Na_2_SO_4_, filtered, and concentrated. The product was then precipitated from solution using diethyl ether and obtained as a white powder (4.5 g, 90%). ^1^H NMR (300 MHz, CDCl_3_, δ): 8.29 (m, 2H; Ar H), 7.41 (m, 2H; Ar H), 4.45 (m, 2H; CH_2_), 3.65 (s, 245H; PEG H). ^1^H NMR spectroscopy indicated 96% conversion of the end group.

NPC-substituted 4aPEG in CH_2_Cl_2_ (50 mL) was added dropwise to N_2_H_4_·H_2_O (0.9 mL, 10 eq) in CH_2_Cl_2_ (50 mL). The mixture was stirred overnight and evaporated in vacuo. The residue was dissolved in deionized water, dialyzed for 2 days, and lyophilized as a white fluffy solid in nearly quantitative yield. ^1^H NMR (300 MHz, CDCl_3_, δ): 4.29 (m, 2H; CH_2_), 3.65 (s, 257H; PEG H). ^1^H NMR spectroscopy indicated 94% conversion of the end group. PDI = 1.20.

**2.5 Synthesis of *o*-Phthalaldehyde (OPA) derivative**

First, 3,4-bis(dibromomethyl)benzoic acid was synthesized from commercially available 3,4-dimethylbenzoic acid according to the previously reported method.^4^ Specifically, 3,4-dimethylbenzoic acid (15 g, 0.1 mol) and *N*-bromosuccinimide (89 g, 0.5 mol, 5eq) were dissolved in warm carbon tetrachloride (300 mL). Benzoyl peroxide (2.4 g, 10 mmol) was added and the reaction mixture was refluxed at 81℃ for 15 h. The white precipitate was filtered in heat and washed with toluene three times (3 × 100 mL), followed by washing with diethyl ether three times (3 × 100 mL). The combined filtrate was evaporated, and the residue was dissolved in 10% Na_2_CO_3_ (350 mL). This solution was washed with CH_2_Cl_2_ and acidified to pH 1 with concentrated HCl and then extracted with EtOAc (4 × 60 mL). The organic phase was washed with brine, dried with Na_2_SO_4_, filtered, and evaporated in vacuo. The pale yellow solid was purified by crystallization in acetonitrile to yield 3,4-bis(dibromomethyl)benzoic acid (18 g, 40%). ^1^H NMR (300 MHz, DMSO-*d*_6_, δ): 13.54 (s, 1H; COOH), 8.35 (s, 1H; Ar H), 8.01 (m, 2H; Ar H), 7.79 (s, 1H; CH), 7.77 (s, 1H; CH).

3,4-bis(dibromomethyl)benzoic acid was then dissolved in 10% Na_2_CO_3_ (180 mL) and kept at 70℃ for 4 h. The reaction mixture was acidified with HCl to pH 1 in an ice-water bath and extracted with EtOAc (5 × 40 mL). The organic layer was washed with brine, dried with Na_2_SO_4_, filtered, and evaporated in vacuo. The yellow solid (5 g) thus obtained was dissolved in anhydrous CH_3_OH (100 mL) and treated with Sc(OTf)_3_ (750 mg) at r.t. overnight. After that, CH_3_OH was evaporated, the mixture was dissolved in CH_3_CN (100 mL), and NHS (4.8 g) and EDC·HCl (8.1 g) were added. The mixture was stirred at r.t. overnight and concentrated in vacuo. The residue was dissolved in CH_2_Cl_2_, washed with brine thrice and dried with MgSO_4_. The crude product was purified by silica gel column chromatography in Hex/EtOAc affording 1,3-dimethoxy-1,3-dihydroisobenzofuran-5-carboxylic acid *N*-succinimidyl ester as a mixture of isomers. ^1^H NMR (300 MHz, CDCl_3_, δ): 8.23 (m, 2H, Ar H), 7.57 (d, 1H; Ar H), 6.25 (m, 2H; CH), 3.48 (m, 6H; CH_3_), 2.94 (s, 4H; NHS H); ^13^C NMR (75 MHz, CDCl_3_, δ): 169.1, 161.3, 145.1, 139.6, 132.3, 126.9, 125.8, 123.8, 106.1, 105.1, 54.8, 25.7. HR-ESI-MS *m/z*: [M + Na]^+^ calcd for C_15_H_15_NO_7_, 344.07407; found, 344.07319.

**2.6 *o*-Phthalaldehyde (OPA) terminated 4-arm PEG (4P-OPA)**

4P-NH_2_ (2 g, 0.8 mmol of NH_2_) and 1,3-dimethoxy-1,3-dihydroisobenzofuran-5-carboxylic acid *N*-succinimidyl ester (514 mg, 1.6 mmol, 2eq) were dissolved in anhydrous CH_2_Cl_2_ (25 mL) and pyridine (0.5 mL) was added. The mixture was stirred at r.t. for 2 days and precipitated in cold diethyl ether to give the product as a pale yellow powder in nearly quantitative yield. ^1^H NMR (300 MHz, CDCl_3_, δ): 7.93 (m, 1H; Ar H), 7.86 (m, 1H; Ar H), 7.47 (m, 1H; Ar H), 6.22 (m, 2H; CH), 3.65 (s, 311H; PEG H). ^1^H NMR spectroscopy indicated 77% conversion of the end group.

(1,3-Dimethoxy-1,3-dihydroisobenzofuran-5-carboxamido)-substituted 4aPEG was then dissolved in deionized water (5 mL) and trifluoroacetic acid (5 mL) was added. The mixture was stirred at r.t. for 1 h and diluted to 50 mL. The mixture was dialyzed for 2 days and lyophilized to afford a white solid of 4P-OPA in nearly quantitative yield. ^1^H NMR (300 MHz, CDCl_3_, δ): 10.61 (s, 1H; CHO H), 10.52 (s, 1H; CHO H), 8.47 (m, 1H; Ar H), 8.28 (m, 1H; Ar H), 8.06 (m, 1H; Ar H), 3.65 (s, 330H; PEG H). ^1^H NMR spectroscopy indicated 73% conversion of the end group. PDI = 1.63.

**3. Gelation behaviors**

**3.1 Vial inversion tests**

The gelation behaviors were determined by vial inversion method. Solutions of 4P-OPA (or 4P-PhCHO) and *N*-nucleophile-terminated 4aPEG at a given concentration in phosphate buffer saline (PBS) were mixed at equimolar ratio of OPA (or PhCHO) to *N*-nucleophile in a glass vial with an inner diameter of 10 mm at 37 ^o^C. The formation of hydrogels was determined if no flow was observed within 30 s after inverting the vial. The critical gelation concentration (CGC) was defined as the lowest polymer concentration required for hydrogel formation within 24 h.

**3.2 Rheological experiments**

Rheological tests were carried out on a rheometer using parallel plates of 25 mm diameter with the gap fixed at 0.5 mm. For gelation kinetics measurements, solutions of 4P-OPA (or 4P-PhCHO) and *N*-nucleophile-terminated 4aPEG in PBS (stored at 0 ^o^C) were mixed at equimolar ratio of OPA (or PhCHO) to *N*-nucleophile and immediately pipetted onto the bottom plate of the rheometer at 37 ^o^C. The storage modulus (*G′*) and loss modulus (*G″*) were recorded as a function of time at a frequency of 1 Hz and strain of 1%. The frequency sweep from 0.1 Hz to 100 Hz was performed at a constant strain of 1%. The strain sweep from 0.1% to 500% was performed at a constant frequency of 1 Hz. The gap was sealed by silicon oil to prevent the evaporation of water.

**4. Compressive and tensile tests**

The compressive test was performed on a texture analyzer (CT3-1000, Brookfield, USA). Solutions of 4P-OPA (or 4P-PhCHO) and 4P-NHNH_2_ were mixed at equimolar ratio of reactive groups and immediately pipetted in cylindrical molds (10 mm in diameter and 5 mm in height). The mixtures were incubated at 37 ^o^C for 6h before the test. The compressive rate was 30 mm/min. All tests were repeated three times.

The tensile test was performed on a universal testing machine (AGS-X 100N, SHIMADZU, Japan). Hydrogels were prepared in rectangular molds as mentioned above (35 mm in length, 5 mm in width and 2 mm in thickness). The tensile rate was 1 mm/min. All tests were repeated three times.

**5. Study of model reaction**

**5.1 Kinetic NMR analysis**

All 1D proton experiments were performed using exactly the same parameters (30 scans, acquisition time of 2 min) and were processed similarly. The same integration regions were used for each spectrum. Chemical shifts and integrals were referenced to the residual solvent peak of DMSO (2.50 ppm). For each spectrum, time points were corrected by half the acquisition time.

Stock solutions for OPA and *N*-nucleophile (methylamine, ethyl carbazate, or ethoxyamine) were freshly prepared in DMSO-*d*_6_. K_3_PO_4_ buffer stock solution was freshly prepared in D_2_O and the pH was adjusted to desired values with concentrated DCl. All experiments were set up as follow: 1) 400 μL of phosphate buffer (1/15 M) was mixed with 50 μL of OPA (100 mM) in an Eppendorf tube. 2) Then, 50 μL of *N*-nucleophile (methylamine, ethyl carbazate, or ethoxyamine) (100 mM) was added, and the mixture was vortexed thoroughly and pipetted into the NMR tube for measurement. The final volume of the NMR sample was 500 μL, and the final concentrations of the solutes included 10 mM of OPA and *N*-nucleophile (methylamine, ethyl carbazate, or ethoxyamine), as well as 53 mM of phosphate buffer in D_2_O/DMSO-*d*_6_ at a volume ratio of 4:1.

**5.2 Characterization of condensation products**

OPA (134 mg, 1 mmol) was dissolved in phosphate buffer (95 mL) at 60℃. After cooling to r.t., *N*-nucleophile (methylamine, ethyl carbazate, or ethoxyamine) (1 mmol) in phosphate buffer (5 mL) was added. The mixture was stirred at r.t. for 90 min and lyophilized to give a white fluffy solid. The condensation products were extracted with CH_2_Cl_2_ and purified by silica gel column chromatography in CH_2_Cl_2_/EtOAc.

The general procedure was followed to give the phthalimidine product as orange solid. ^1^H NMR (300 MHz, DMSO-*d*_6_, δ): 7.66 (dd, *J* = 7.6, 1.1 Hz, 1H), 7.58 (m, 2H), 7.47 (dq, *J* = 8.0, 4.1 Hz, 1H), 4.45 (s, 2H), 3.07 (s, 3H); ^13^C NMR (126 MHz, DMSO-*d*_6_, δ): 167.26, 141.71, 132.44, 131.09, 127.74, 123.25, 122.55, 51.35, 28.93; ESI-MS *m/z*: [M + H]^+^ calcd for C_9_H_9_NO, 148.1; found, 147.9.


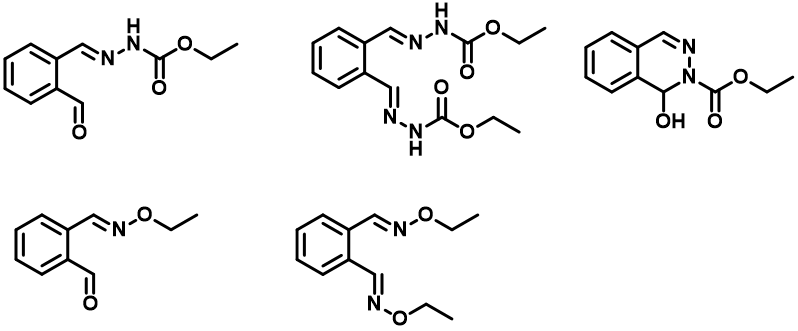


The general procedure was followed to give the hydrazone product as white solid. ^1^H NMR (300 MHz, DMSO-*d*_6_, δ): 10.22 (s, 1H), 8.78 (s, 1H), 7.92 (dd, *J* = 7.5, 1.5 Hz, 2H), 7.66 (dtd, *J* = 26.1, 7.5, 1.4 Hz, 2H), 4.16 (q, *J* = 7.1 Hz, 2H), 1.24 (t, *J* = 7.1 Hz, 3H); ^13^C NMR (126 MHz, DMSO-*d*_6_, δ): 194.00, 153.93, 141.88, 135.41, 134.14, 134.03, 132.84, 130.01, 127.73, 61.14, 15.02; ESI-MS *m/z*: [M + H]^+^ calcd for C_11_H_12_N_2_O_3_, 221.1; found, 221.1.


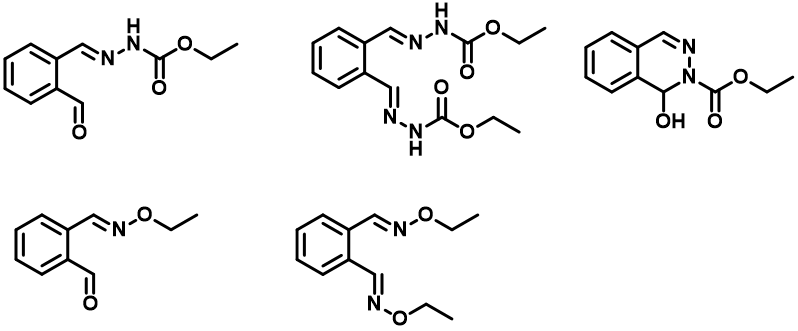


The general procedure was followed to give the cyclization product as colorless oil. ^1^H NMR (300 MHz, DMSO-*d*_6_, δ): 7.96 (s, 1H), 7.54 (m, 4H), 6.61 (m, 2H), 4.26 (q, *J* = 7.1, 1.0 Hz, 2H), 1.29 (t, *J* = 7.1 Hz, 3H); ^13^C NMR (126 MHz, DMSO-*d*_6_, δ): 154.05, 140.96, 132.02, 131.33, 129.15, 127.04, 125.62, 123.24, 71.50, 62.13, 14.39; ESI-MS *m/z*: [M + H]^+^ calcd for C_11_H_12_N_2_O_3_, 221.1; found, 221.3.


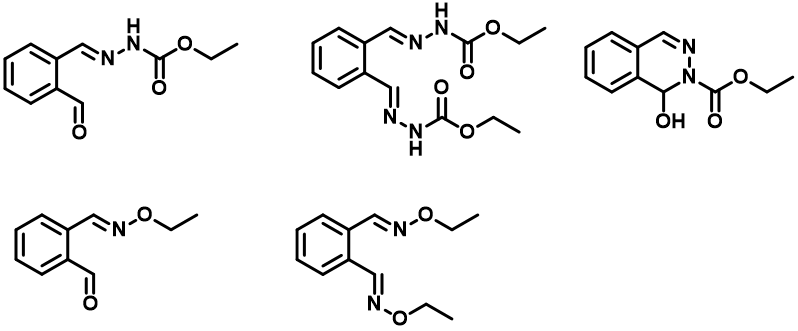


The general procedure was followed to give the bis-hydrazone product as white solid. ^1^H NMR (300 MHz, DMSO-*d*_6_, δ): 8.38 (s, 2H), 7.74 (dt, *J* = 7.2, 3.6 Hz, 2H), 7.43 (dd, *J* = 5.9, 3.3 Hz, 2H), 4.15 (q, *J* = 7.1 Hz, 4H), 1.24 (t, *J* = 7.1 Hz, 6H); ^13^C NMR (126 MHz, DMSO-*d*_6_, δ): 153.95, 142.28, 132.93, 129.78, 127.62, 61.04, 15.03; ESI-MS *m/z*: [M + H]^+^ calcd for C_14_H_18_N_4_O_4_, 307.1; found, 307.3.


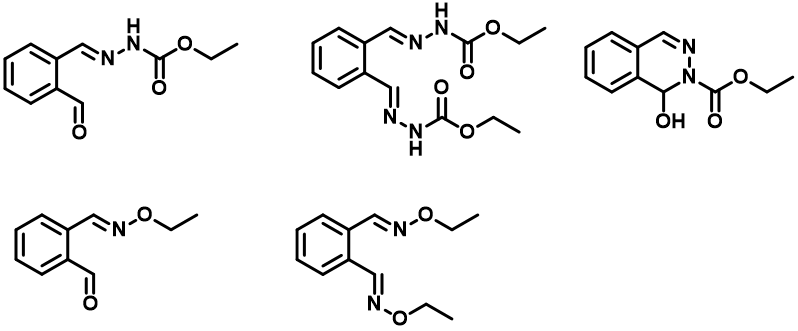


The general procedure was followed to give the oxime product as colorless oil. ^1^H NMR (300 MHz, DMSO-*d*_6_, δ): 10.23 (s, 1H), 8.87 (s, 1H), 7.89 (ddd, *J* = 24.0, 7.5, 1.5 Hz, 2H), 7.68 (dtd, *J* = 18.7, 7.3, 1.5 Hz, 2H), 4.20 (q, *J* = 7.0 Hz, 2H), 1.27 (t, *J* = 7.0 Hz, 3H); ^13^C NMR (126 MHz, DMSO-*d*_6_, δ): 193.74, 146.90, 134.23, 134.19, 132.96, 132.12, 130.47, 128.17, 69.96, 14.96; ESI-MS *m/z*: [M + H]^+^ calcd for C_11_H_13_NO_2_, 178.1; found, 178.0.

**5.3 Kinetic analysis**

The kinetics of the reaction between OPA and methylamine were studied by recording UV-vis absorption with a UV-vis spectrometer (PerkinElmer Inc., USA). The reaction was performed under second-order conditions in quartz cuvettes (10 mm path length, total volume of 2 mL) at 23 ℃. Stock solutions of OPA and methylamine were freshly prepared prior to measurement. 200 μL of OPA stock solution (1 mM in PBS) was added to an Eppendorf tube with 1600 μL of PBS, followed by addition of 200 μL of methylamine stock solution (1 mM in PBS). The mixture was vortexed thoroughly and pipetted into the quartz cuvettes. The final volume of the solution was 2 mL, and the final concentrations of the solutes included 0.1 mM of OPA and 0.1 mM of methylamine. UV-vis spectra were recorded on 180 second intervals. A shift of absorption maxima from 259 nm to 238 nm was observed during the measurement. The kinetic data were fitted to a second-order kinetic equation shown below to obtain the rate constant.^5^ The experiment was repeated three times.

$$-\frac{dc}{dt}=k_{2}c^{2}$$

where *c* is the concentration of OPA and methylamine. Integrate the equation to obtain:

$$\frac{1}{c}-\frac{1}{c_{0}}=k_{2}t$$

where *c*_0_ is the initial concentration of OPA and methylamine. The *k*_2_ value was provided by plotting 1/*c* versus time and fitting the kinetic data according to the linear equation.

6**. Cytotoxicity evaluation**

**6.1 Cell viability**

Mouse fibroblast L929 cells were cultured in Dulbecco’s modified Eagle’s medium (DMEM) containing 10% fetal bovine serum, supplemented with 50 U mL^-1^ penicillin and 50 U mL^-1^ streptomycin and incubated at 37℃ in 5% CO_2_ atmosphere.

Harvested L929 cells were seeded in 96-well plates at a density of 5,000 cells per well and incubated for 24 h. Fresh media containing polymers at different concentrations were added. After incubation for 48 h, the media were replaced by fresh DMEM, followed by addition of 20 μL of MTT (5 mg/mL). After incubation at 37℃ for 4 h, the precipitated formazan was dissolved in 150 μL of DMSO and the absorbance value at 490 nm was measured on a microplate reader (ELx680, BioTek Instrument Inc., USA). Polyethyleneimine (PEI) 25k (Sigma) was used as the positive control. Cell viability (%) was calculated by comparing the absorbance values with those of PBS wells (n = 6).

**6.2 Cell encapsulation**

Harvested L929 cells were mixed homogeneously with 100 μL of 4P-NHNH_2_ in PBS in a 48-well culture plate by gentle pipetting. 100 μL of 4P-OPA in PBS was added. The total polymer concentration was 2% (w/v) with equimolar ratio of reactive groups. The cell-encapsulated hydrogels were rapidly formed within a few minutes. The viability of L929 cells encapsulated in the hydrogels was determined by a live-dead cell staining kit. In brief, L929 cells-encapsulated hydrogels were incubated in PBS (500 μL) containing 2 μM calcein-AM and 4.5 μM propidium iodine (PI) at 37℃ for 1 h. Cells were then imaged using a fluorescence microscope. Viable cells were stained green with calcein, while dead cells were stained red with PI.

**7. RGD modification and cell adhesion**

Mouse fibroblast NIH 3T3 cells were cultured in Dulbecco’s modified Eagle’s medium (DMEM) containing 10% fetal bovine serum, supplemented with 50 U mL^-1^ penicillin and 50 U mL^-1^ streptomycin and incubated at 37℃ in 5% CO_2_ atmosphere.

4P-OPA was mixed with 4P-NHNH_2_ and 2 mM of c(RGDfK) in a 48-well culture plate. The total polymer concentration was 3% (w/v) with equimolar ratio of reactive groups. The mixture was incubated for 1 h at 37 ℃ to allow the completion of reaction. NIH 3T3 cells in 1 mL of DMEM (2×10^4^ cells/mL) were then seeded on the hydrogel surface. At desired time points, the proliferation of cells was assessed by cell counting kit-8 (CCK-8) method. Briefly, 0.5 mL of CCK-8 solution (10% (v/v) in DMEM medium) was added into each well and incubated for 2 h. The absorbance values at 450 nm were measured by a microplate reader (Tecan Group Ltd., Switzerland). The experiments were performed in triplicate.

NIH 3T3 cells (1×10^5^) were seeded on the surface of the hydrogel with or without RGD modification in a glass bottom plate and cultured for 12 h. The media were removed and washed with PBS. The cells were fixed with paraformaldehyde (4% (w/v)) for 15 min and permeated with Triton X-100 (0.1% (v/v)) for 15 min. F-actin was stained with Alexa Fluor 488 phalloidin (0.5% (v/v)) for 45 min and cell nucleus was stained with DAPI (0.1% (v/v)) for 5 min. The stained cells were observed with a confocal laser scanning microscope (LSM 700, Carl Zeiss AG, Germany).

**8. *In vitro* hydrogel degradation**

Hydrogel components were mixed in vials at polymer concentrations of 5% (w/v) with the molar ratio of functional group at 1:1 to form hydrogel disks (10 mm diameter, 0.4 mL). 3 mL of degradation medium (10 mM PBS, pH = 4.0, 7.4) was then added on top of the hydrogel. At a predetermined time, the degradation medium was removed, and the remaining weight was measured.

**9. Statistical analysis**

The experimental data for the compressive and tensile tests, *in vitro* degradation, and cell studies are provided as mean ± standard deviation (SD). The statistical significance of the differences between different groups was analyzed by the One-Way ANOVA method.

**References:**

1. Yang Y, Zhang J, Liu Z *et al*. Tissue-Integratable and Biocompatible Photogelation by the Imine Crosslinking Reaction. *Adv Mater* 2016; **28**: 2724-30.

2. Wu X, He C, Wu Y *et al*. Synergistic therapeutic effects of Schiffs base cross-linked injectable hydrogels for local co-delivery of metformin and 5-fluorouracil in a mouse colon carcinoma model. *Biomaterials* 2016; **75**: 148-62.

3. Mongondry P, Bonnans-Plaisance C, Jean M *et al*. Mild Synthesis of Amino-Poly(ethylene glycol)s. Application to Steric Stabilization of Clays. *Macromol Rapid Comm* 2003; **24**: 681-5.

4. Statsuk AV, Maly DJ, Seeliger MA *et al*. Tuning a Three-Component Reaction For Trapping Kinase Substrate Complexes. *J Am Chem Soc* 2008; **130**: 17568-74.

5. Bandyopadhyay A, Cambray S and Gao J. Fast Diazaborine Formation of Semicarbazide Enables Facile Labeling of Bacterial Pathogens. *J Am Chem Soc* 2017; **139**: 871-8.


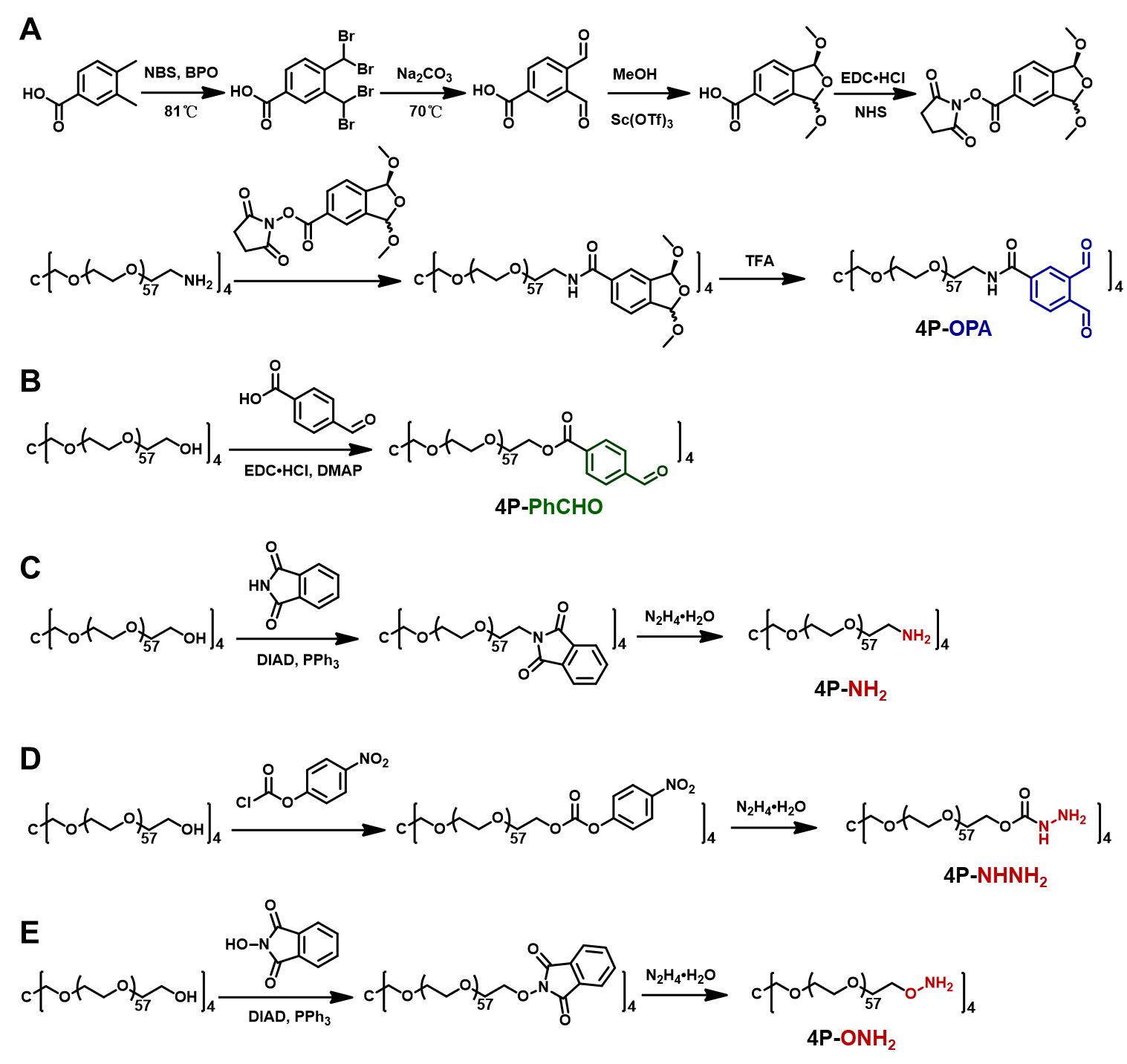


**Scheme S1.** Synthesis routes for A) OPA-terminated 4aPEG (4P-OPA), B) benzaldehyde-terminated 4aPEG (4P-PhCHO), C) primary amine-terminated 4aPEG (4P-NH_2_), D) hydrazide-terminated 4aPEG (4P-NHNH_2_), and E) aminooxy-terminated 4aPEG (4P-ONH_2_), respectively.


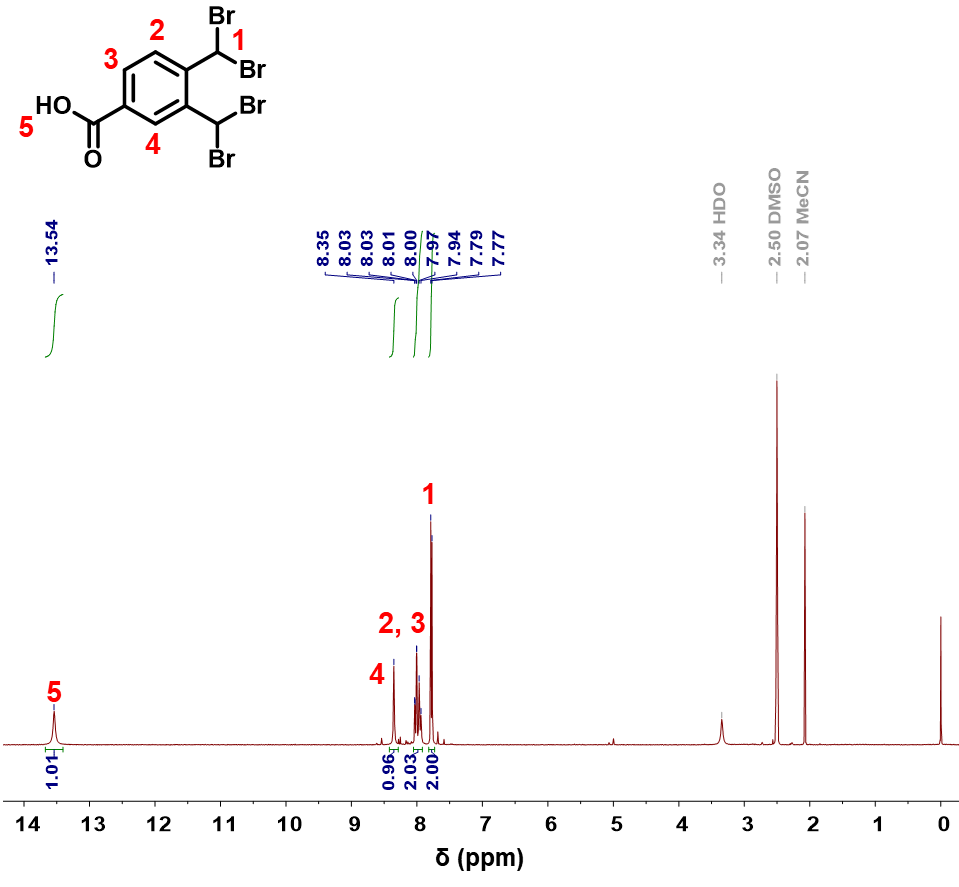


**Figure S1.** ^1^H NMR spectrum of 3,4-bis(dibromomethyl)benzoic acid in DMSO-*d*_6_.


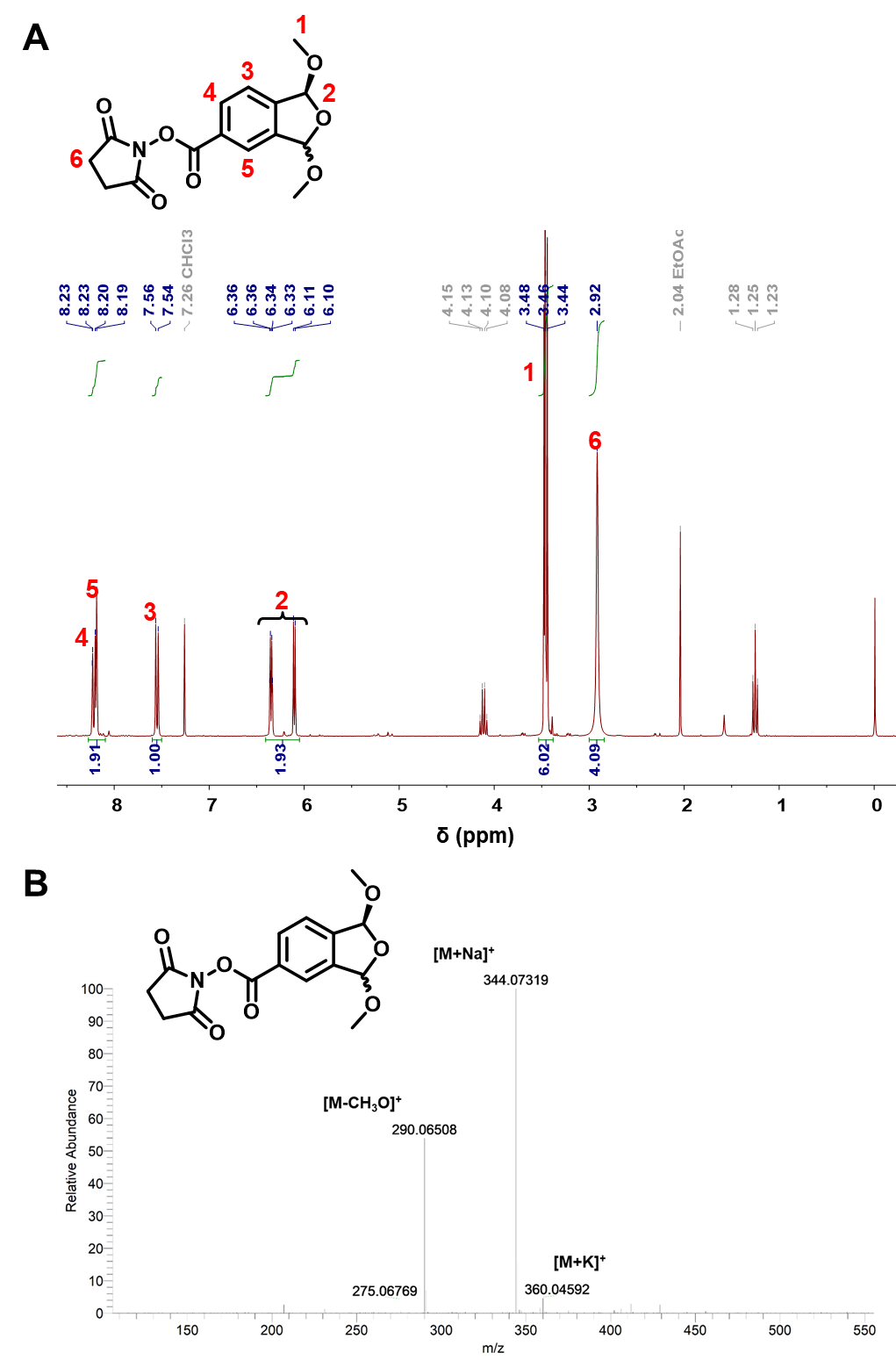


**Figure S2.** (A) ^1^H NMR spectrum of 1,3-dimethoxy-1,3-dihydroisobenzofuran-5-carboxylic acid *N*-succinimidyl ester in CDCl_3_. (B) HR-ESI-MS spectrum of 1,3-dimethoxy-1,3-dihydroisobenzofuran-5-carboxylic acid *N*-succinimidyl ester.


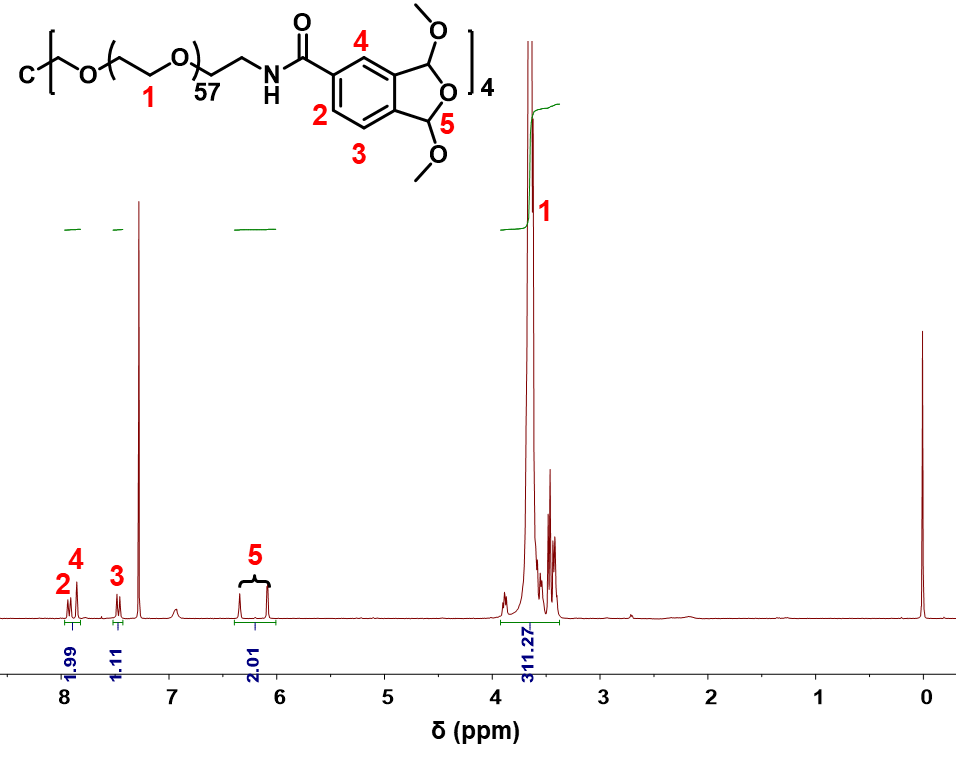


**Figure S3.** ^1^H NMR spectrum of 4aPEG terminated with (1,3-dimethoxy-1,3-dihydroisobenzofuran-5-carboxamido) in CDCl_3_.


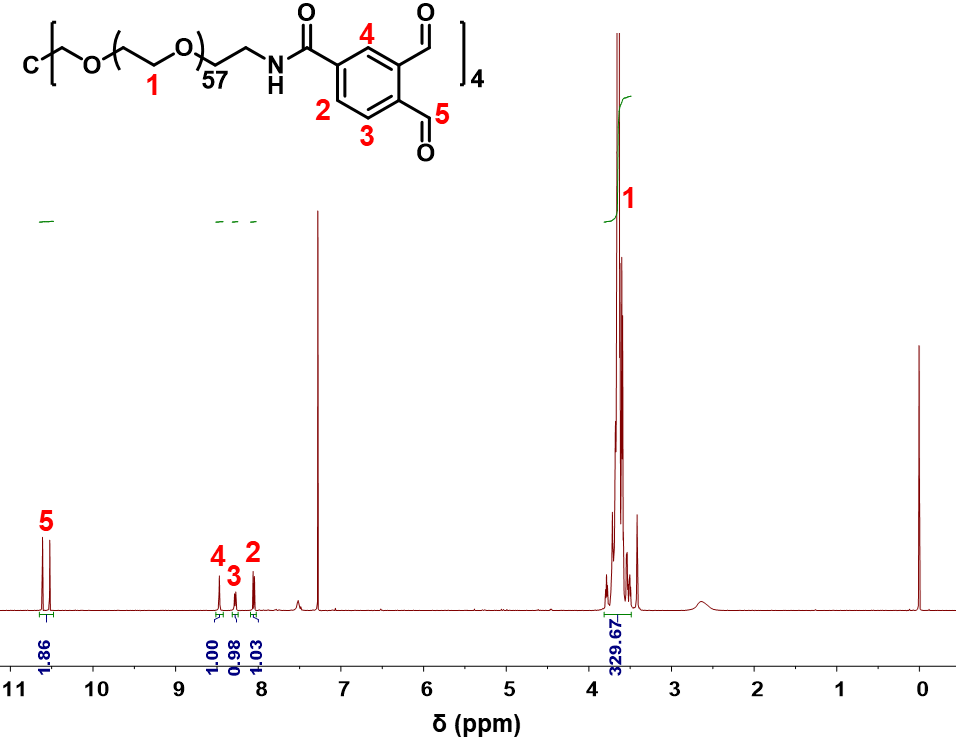


**Figure S4.** ^1^H NMR spectrum of 4P-OPA in CDCl_3_.


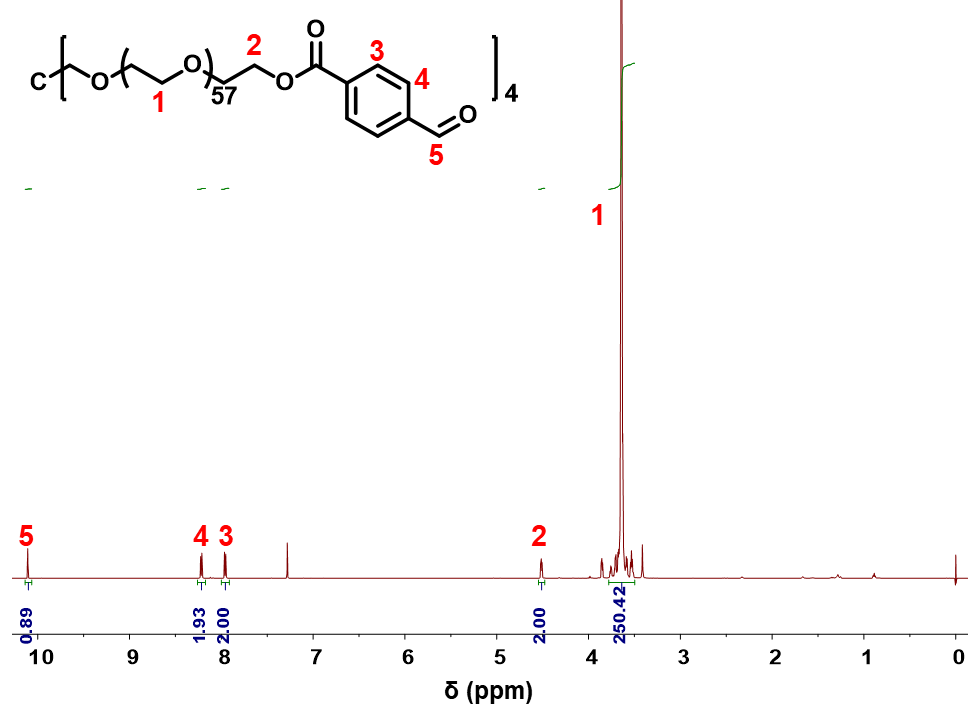


**Figure S5.** ^1^H NMR spectrum of 4P-PhCHO in CDCl_3_.


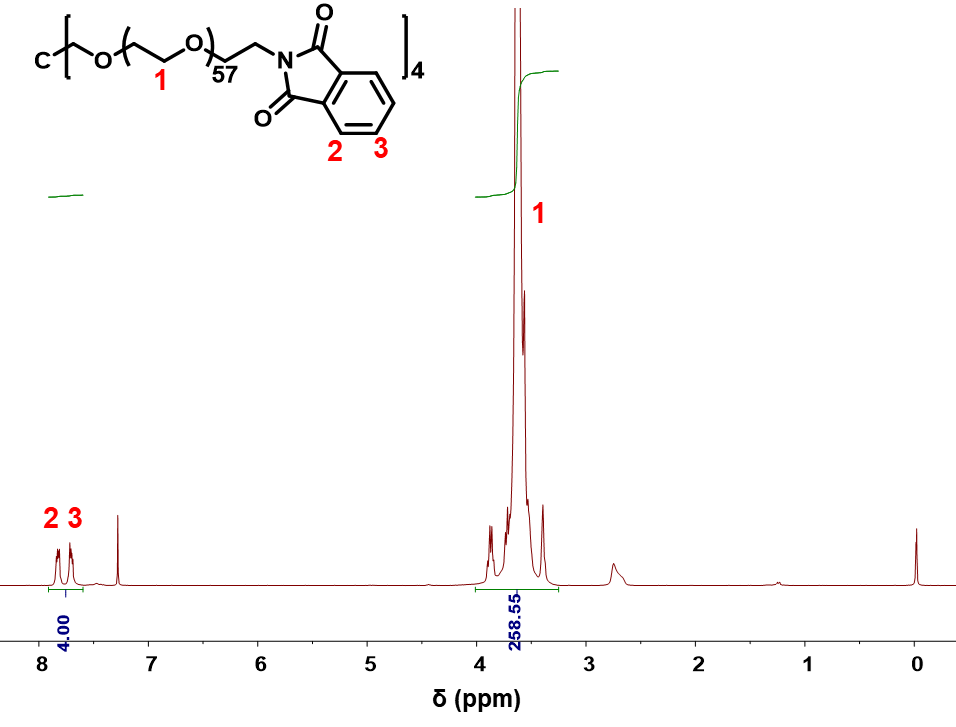


**Figure S6.** ^1^H NMR spectrum of phthalimido-substituted 4aPEG in CDCl_3_.


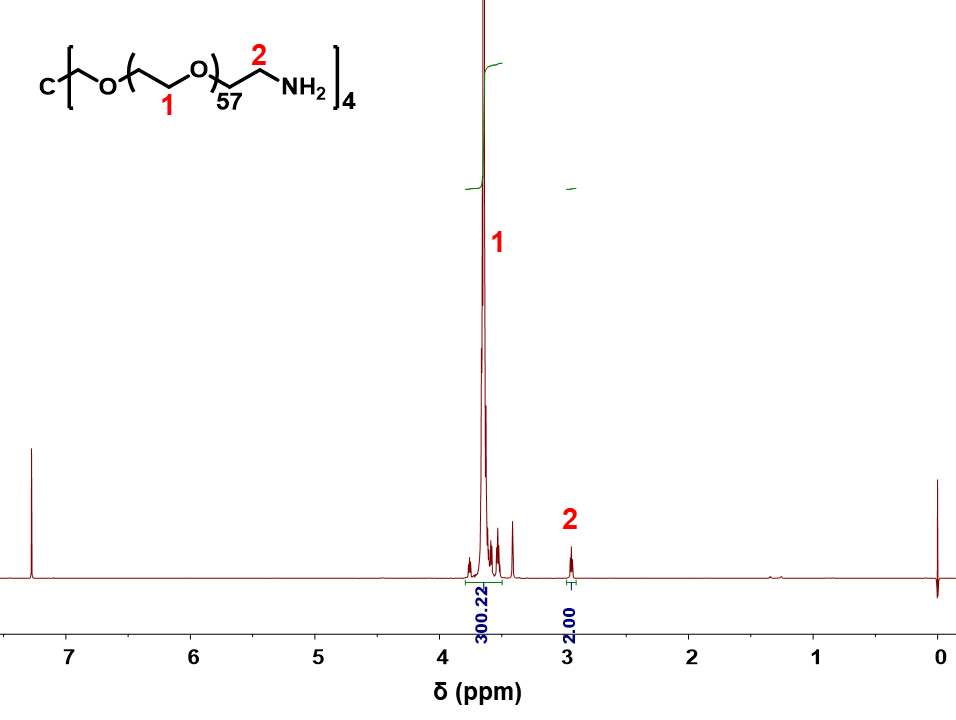


**Figure S7.** ^1^H NMR spectrum of 4P-NH_2_ in CDCl_3_.


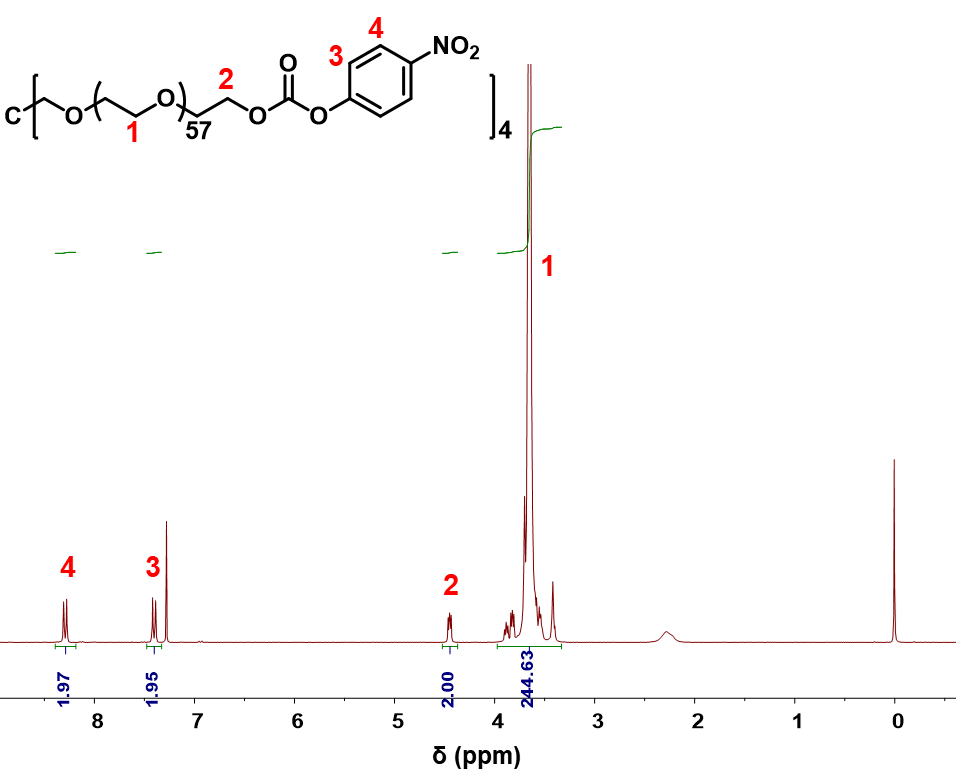


**Figure S8.** ^1^H NMR spectrum of NPC-substituted 4aPEG in CDCl_3_.


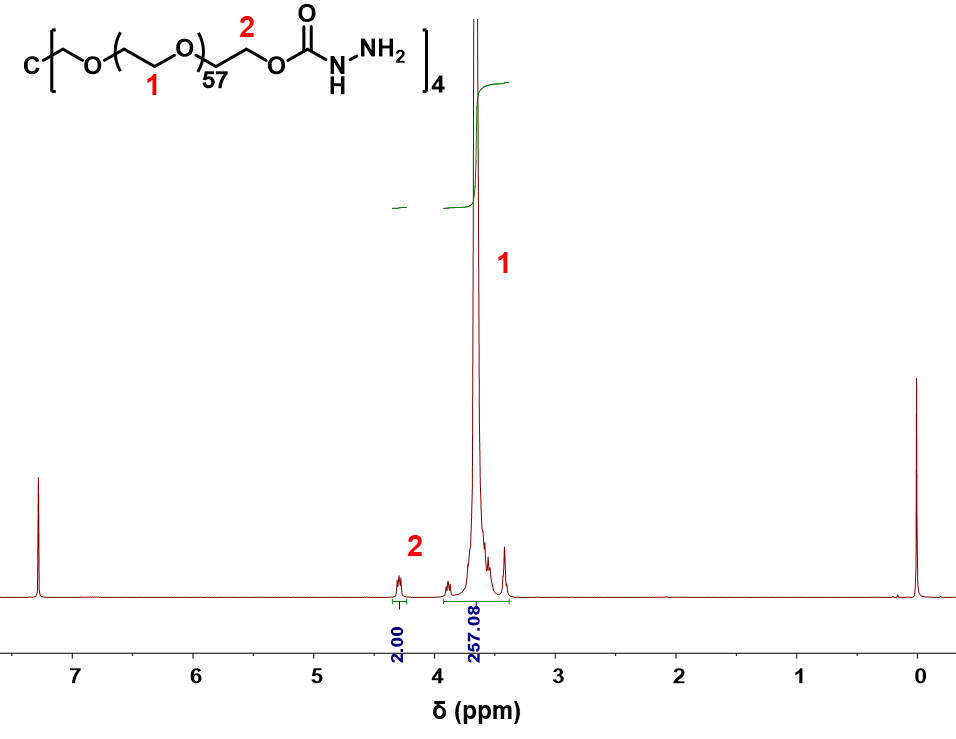


**Figure S9.** ^1^H NMR spectrum of 4P-NHNH_2_ in CDCl_3_.


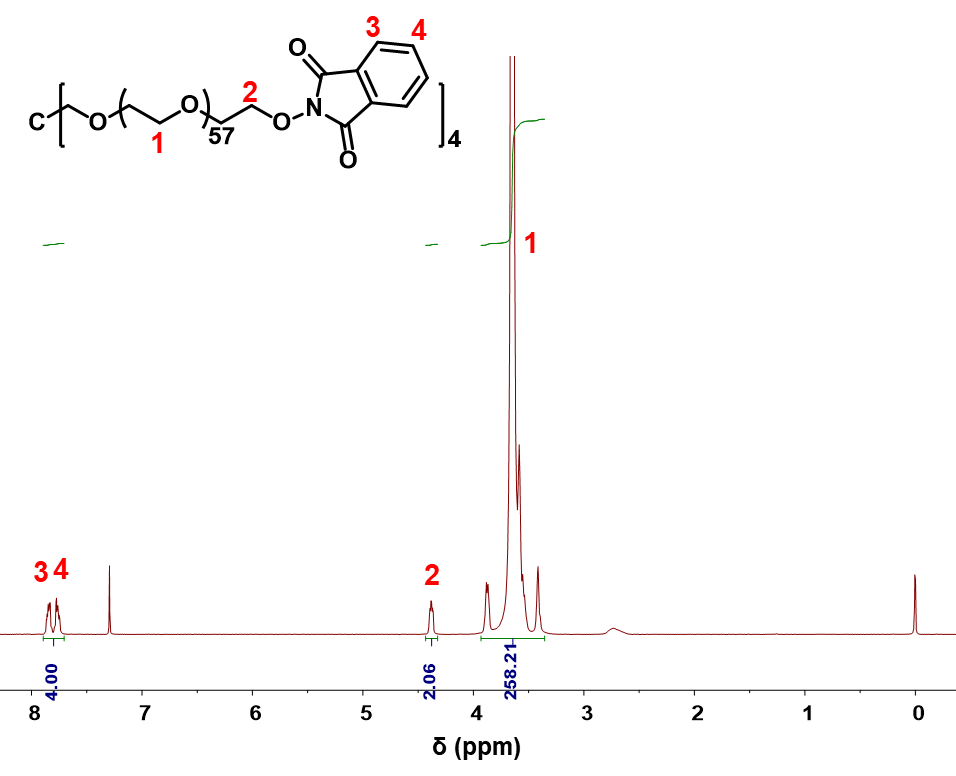


**Figure S10.** ^1^H NMR spectrum of phthalimidooxy-substituted 4aPEG in CDCl_3_.


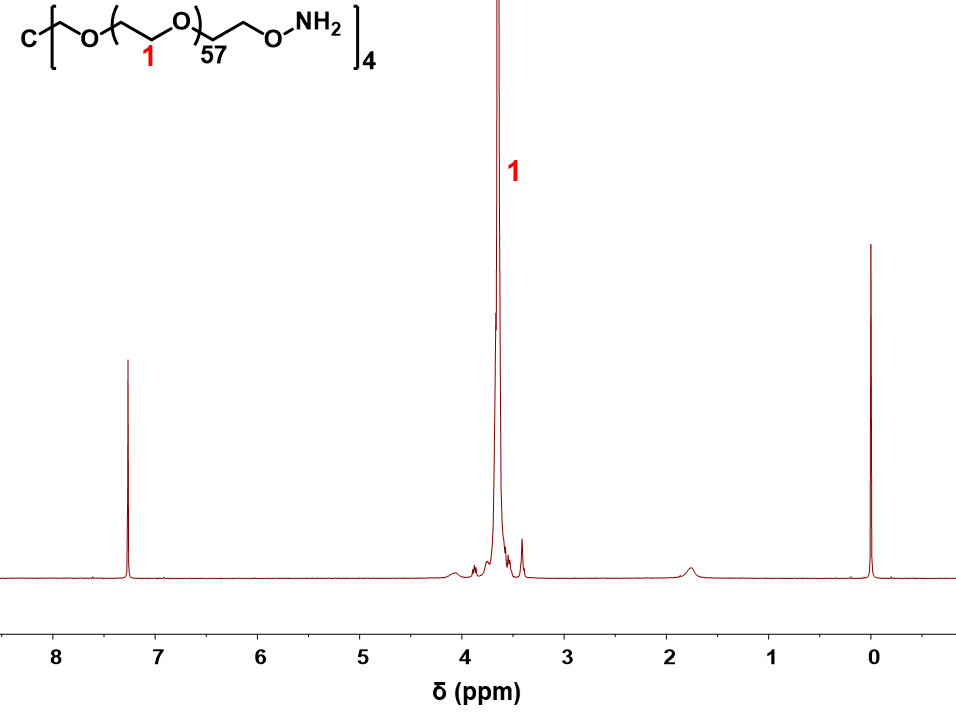


**Figure S11.** ^1^H NMR spectrum of 4P-ONH_2_ in CDCl_3_.

**
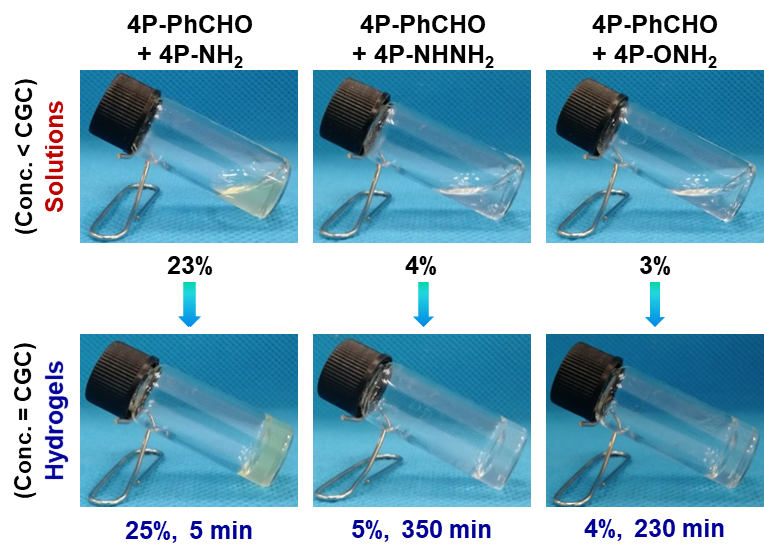
**

**Figure S12.** Photographs showing the solution or hydrogel status of the mixtures of 4P-PhCHO with 4P-NH_2_, 4P-NHNH_2_ and 4P-ONH_2_, respectively, when the polymer concentrations were below or equal to the critical gelation concentrations (CGCs).


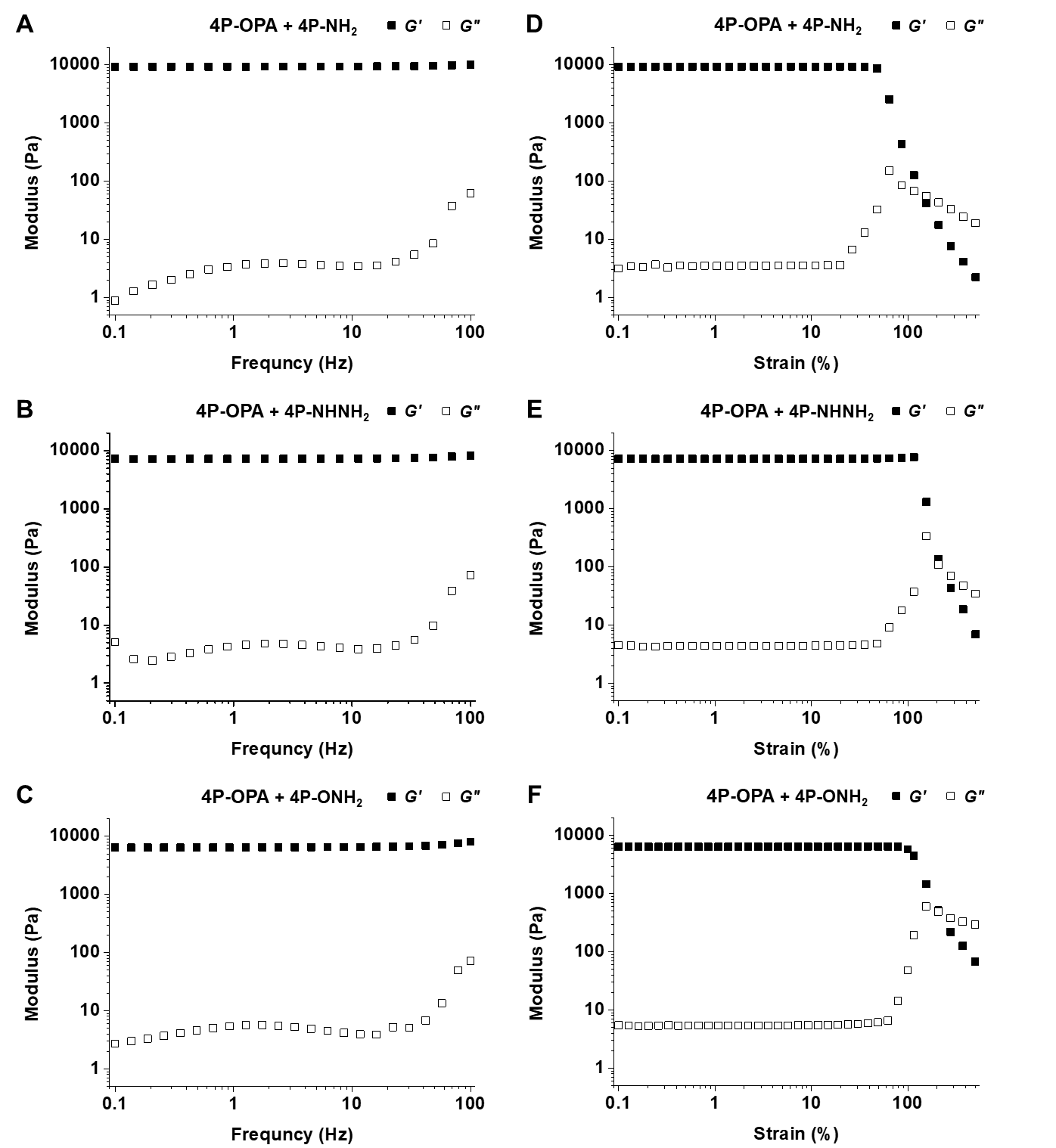


**Figure S13.** Frequency sweep of (A) 4P-OPA/4P-NH_2_, (B) 4P-OPA/4P-NHNH_2_, and (C) 4P-OPA/4P-ONH_2_ hydrogels (5% (w/v)) at a constant strain of 1%. Strain sweep of (D) 4P-OPA/4P-NH_2_, (E) 4P-OPA/4P-NHNH_2_, and (F) 4P-OPA/4P-ONH_2_ hydrogels (5% (w/v)) at a constant frequency of 1 Hz.


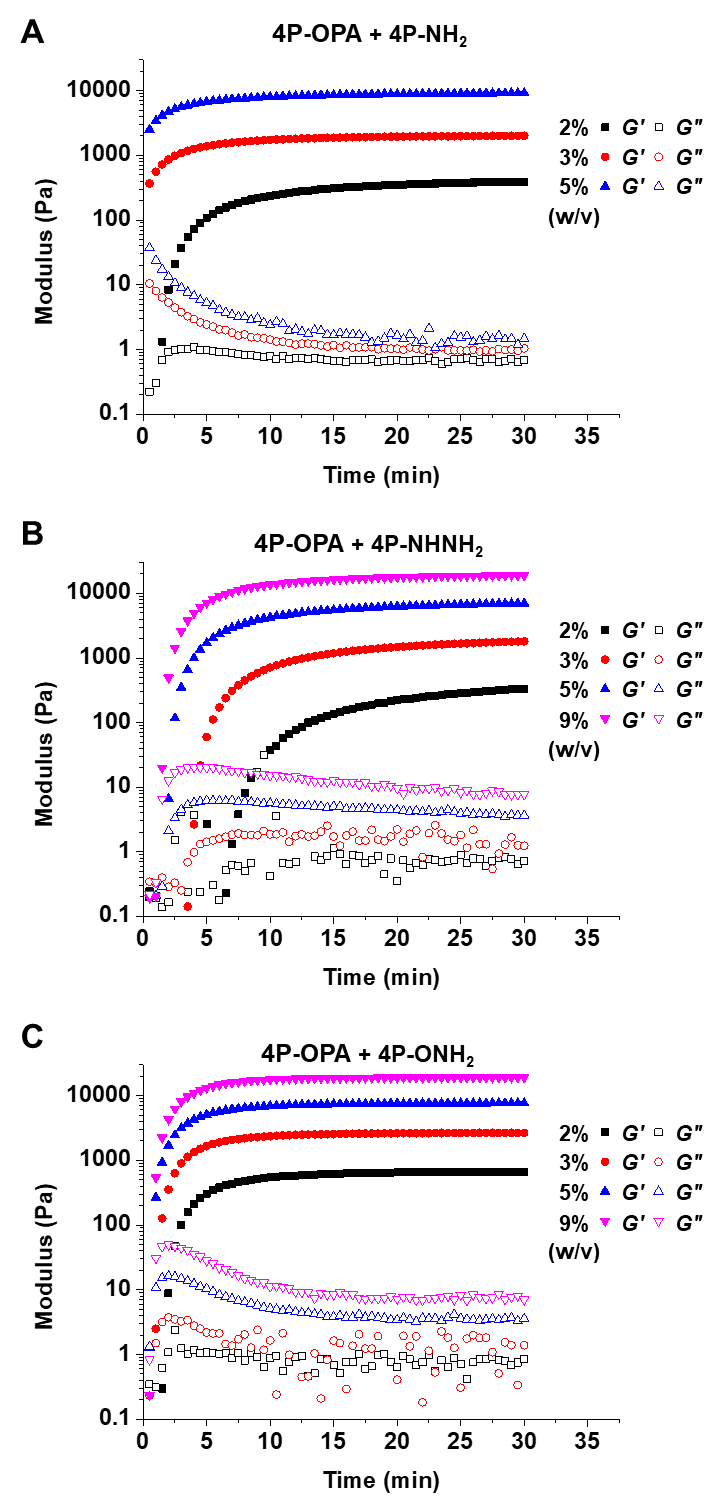


**Figure S14.** Time sweep rheological measurement for the mixtures of (A) 4P-OPA with 4P-NH_2_, (B) 4P-OPA with 4P-NHNH_2_, or (C) 4P-OPA with 4P-ONH_2_ at different polymer concentrations. All the tests were performed at pH 7.4 and 37 ^o^C.


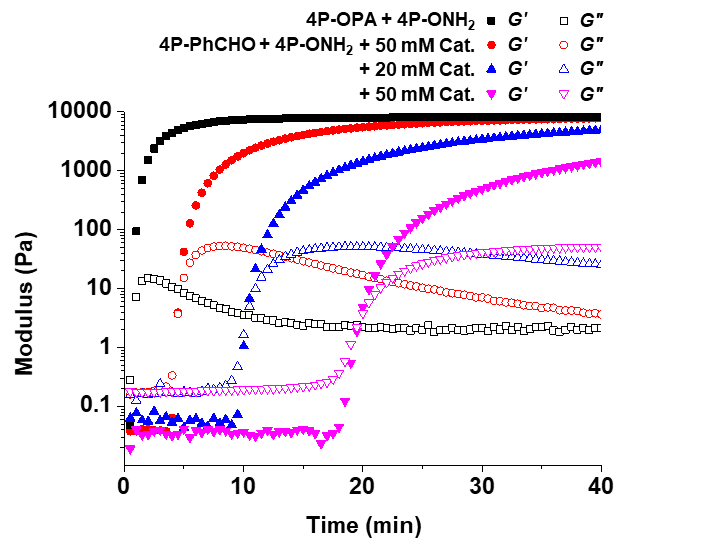


**Figure S15.** Time sweep rheological measurement for the 5% (w/v) mixtures of 4P-OPA with 4P-ONH_2_, compared with those for the 5% (w/v) mixtures of 4P-PhCHO with 4P-ONH_2_ in the presence of 10 ~ 50 mM of aniline catalyst.


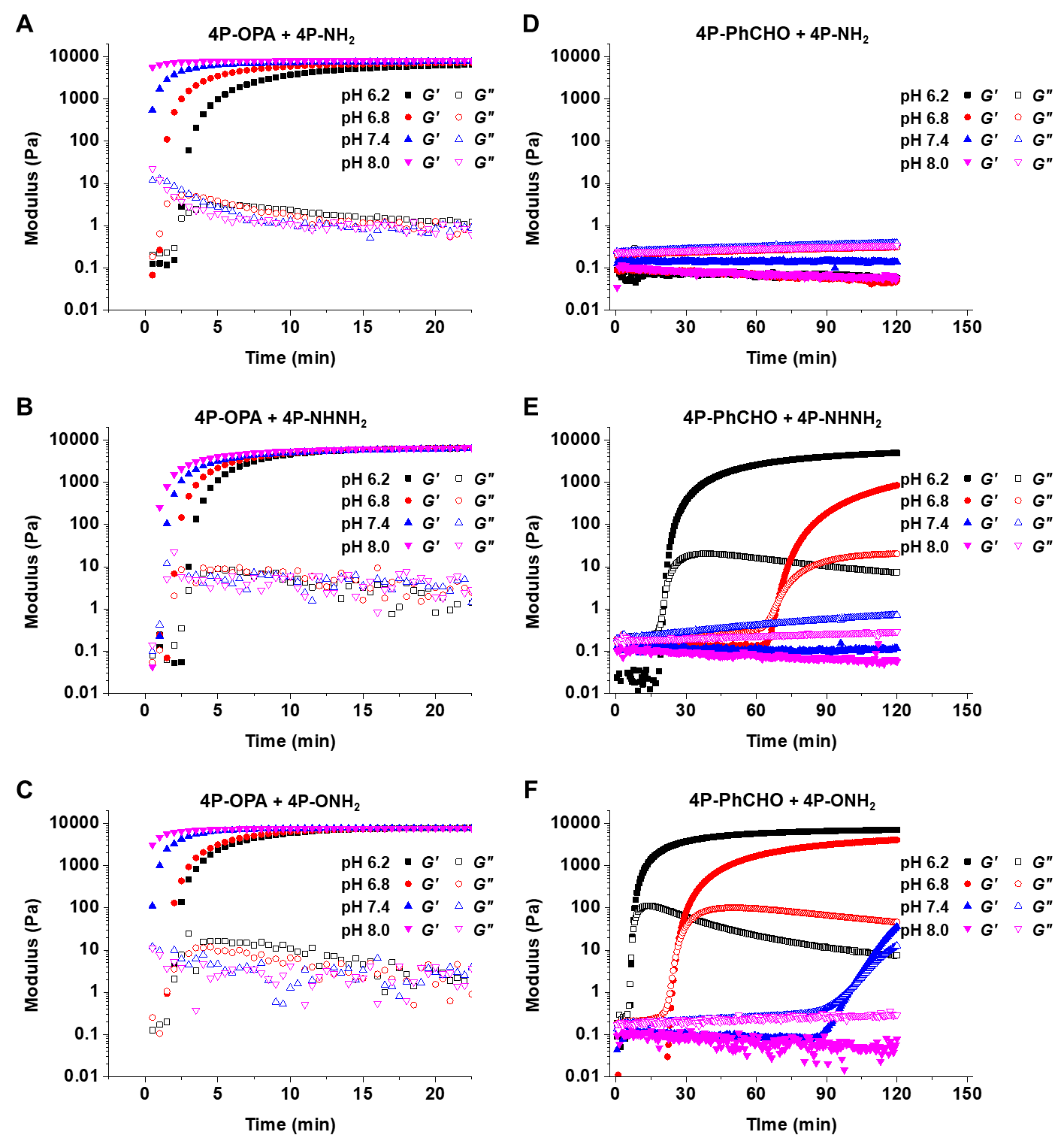


**Figure S16.** Time sweep rheological measurement at various pH for the 5% (w/v) mixtures of (A) 4P-OPA with 4P-NH_2_, (B) 4P-OPA with 4P-NHNH_2_, (C) 4P-OPA with 4P-ONH_2_, (D) 4P-PhCHO with 4P-NH_2_, (E) 4P-PhCHO with 4P-NHNH_2_, and (F) 4P-PhCHO with 4P-ONH_2_ at 37 ^o^C.


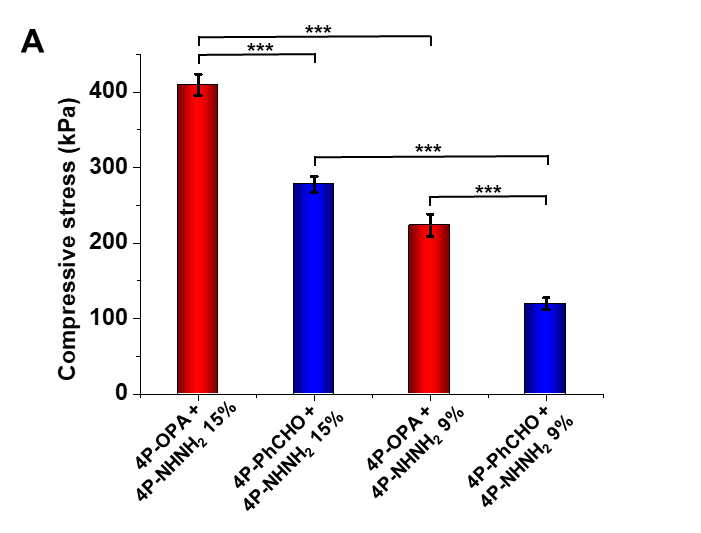


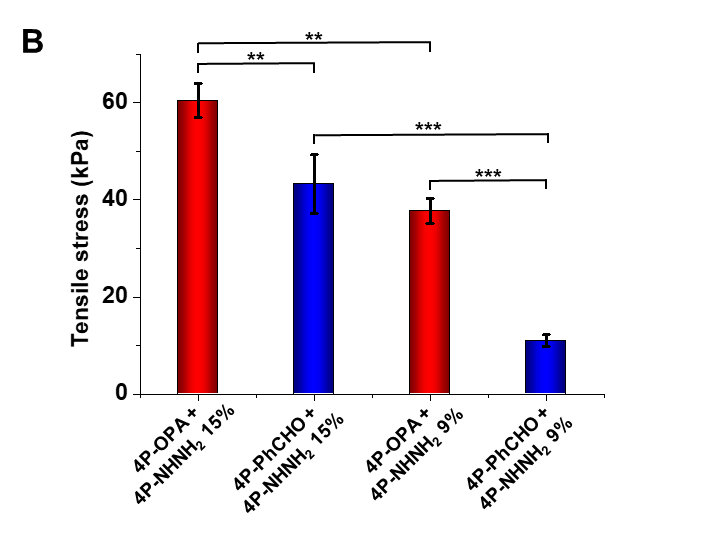


**Figure S17.** (A) Compressive and (B) tensile modulus of different hydrogels. The results are presented as mean ± standard deviation (mean ± SD, ***p* < 0.01, ****p* < 0.001, One-Way ANOVA, n = 3).


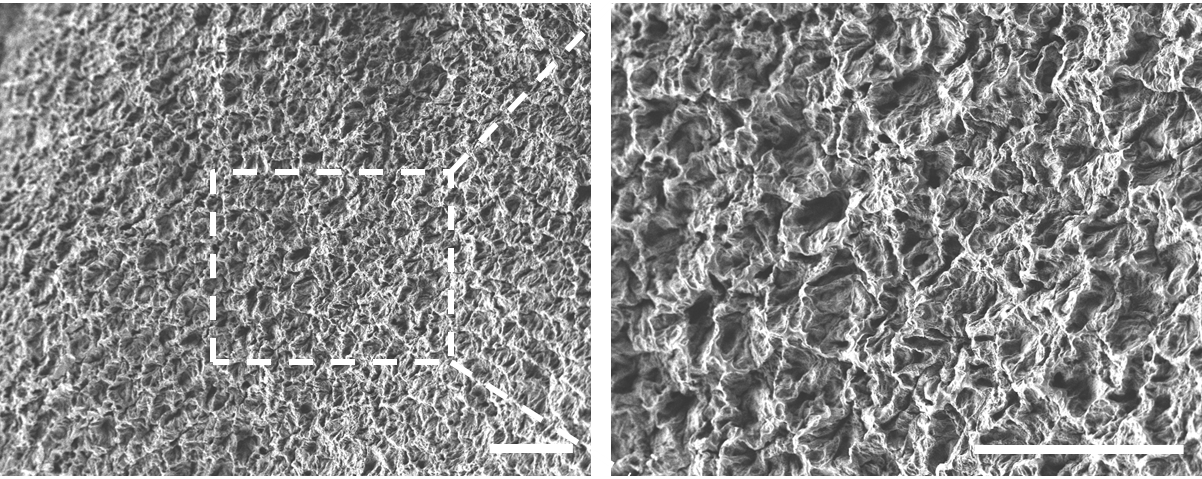


**Figure S18.** Representative SEM images of 4P-OPA/4P-NHNH_2_ hydrogels at polymer concentration of 9% (w/v). Scale bar: 60 μm.


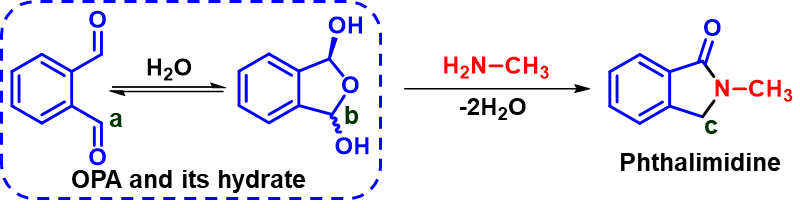


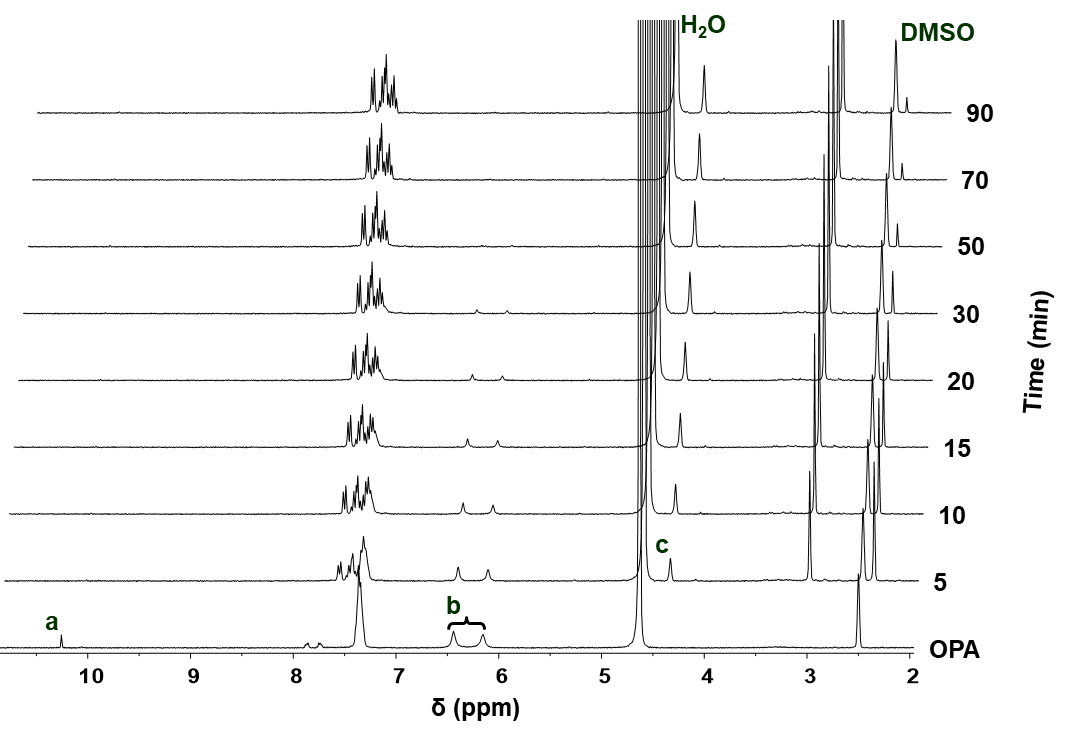


**Figure S19.** Time-dependent ^1^H NMR analysis of the reaction of OPA with methylamine in D_2_O/DMSO-*d*_6_ (4:1 (v/v)).


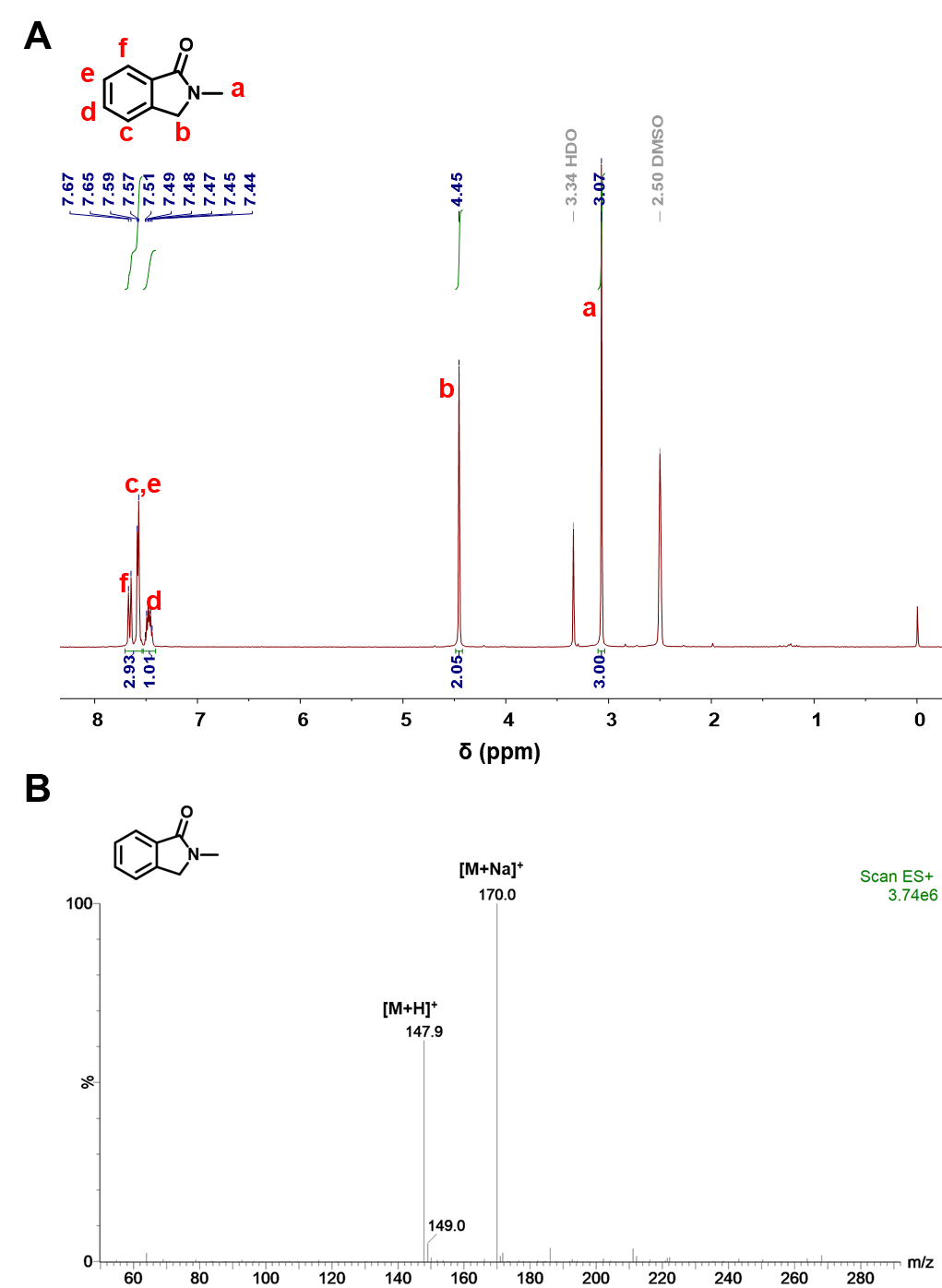


**Figure S20.** (A) ^1^H NMR spectrum of phthalimidine in DMSO-*d*_6_. (B) ESI-MS spectrum of phthalimidine.

**
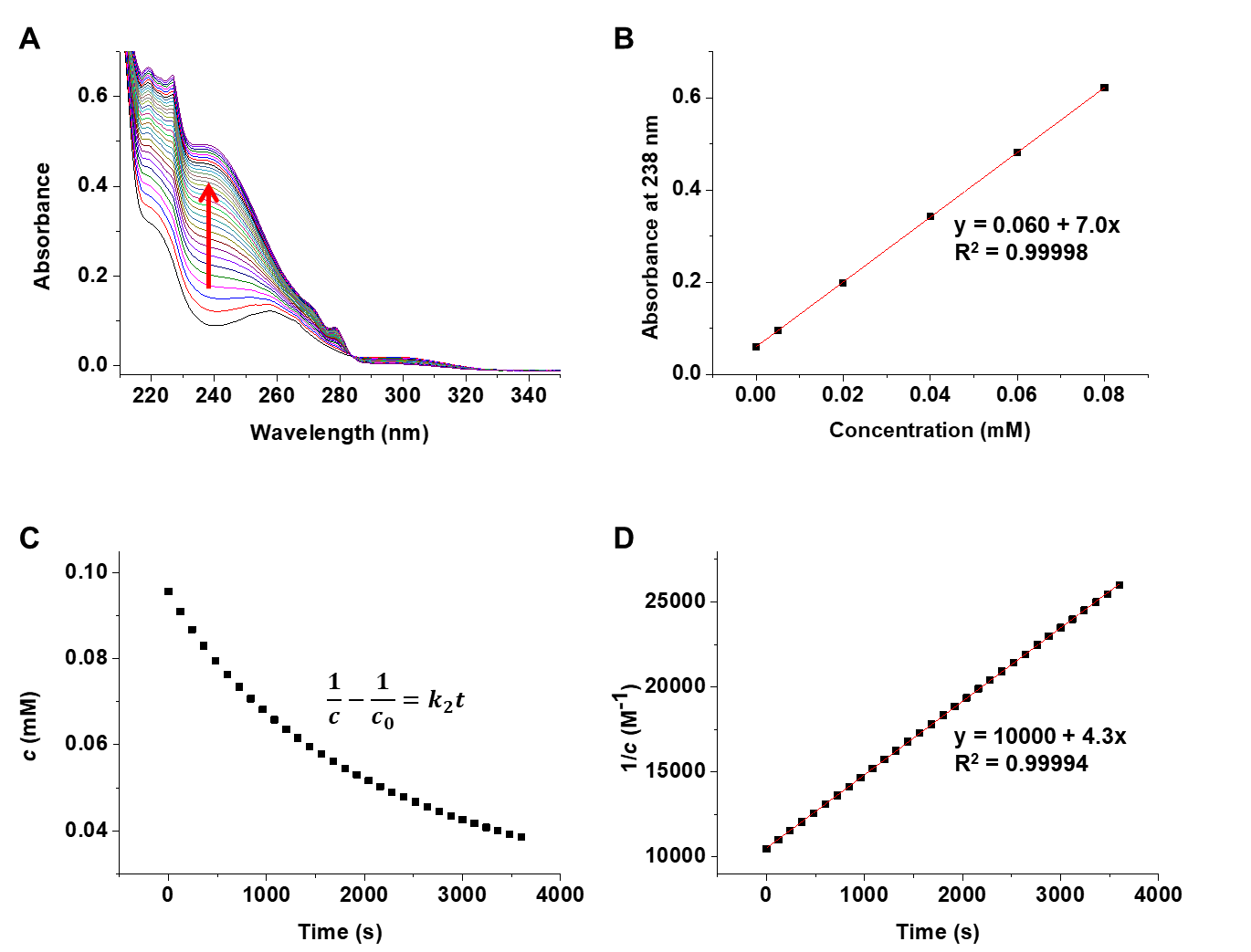
**

**Figure S21.** Determination of second-order rate constant of phthalimidine formation from OPA and methylamine in PBS. (A) Kinetic profile of phthalimidine formation monitored by UV-vis spectrometry. (B) Standard curve used to determine the concentration of phthalimidine and residual OPA. (C) Concentration of OPA over time. (D) Second-order plot for kinetic data. The second-order rate constant was provied as the slope of the linear fit.


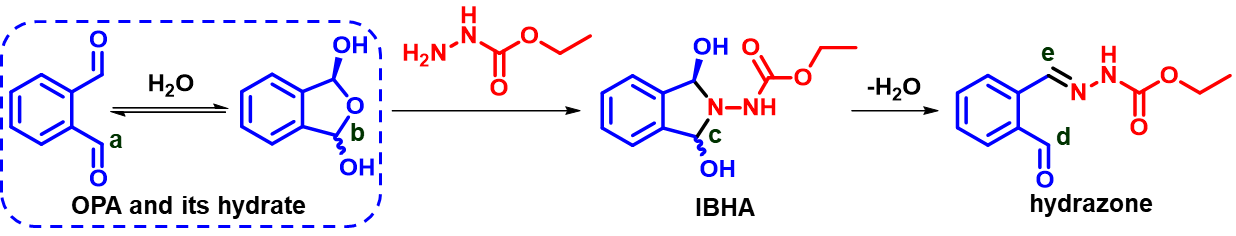


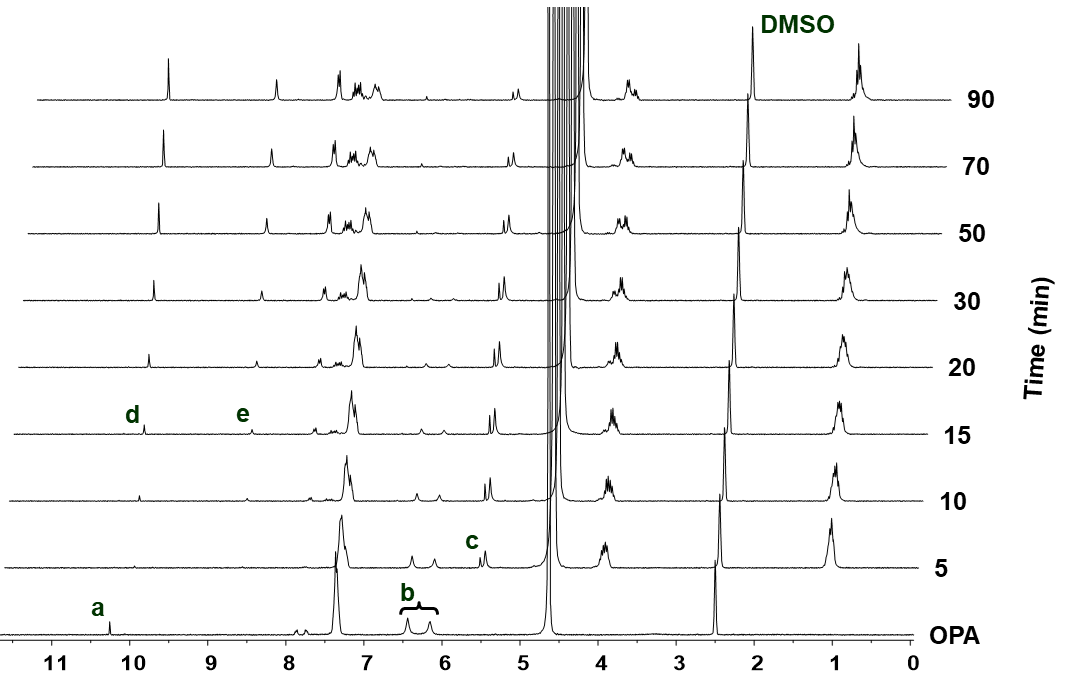


**Figure S22.** Time-dependent ^1^H NMR analysis of the reaction of OPA with ethyl carbazate in D_2_O/DMSO-*d*_6_ (4:1 (v/v)).


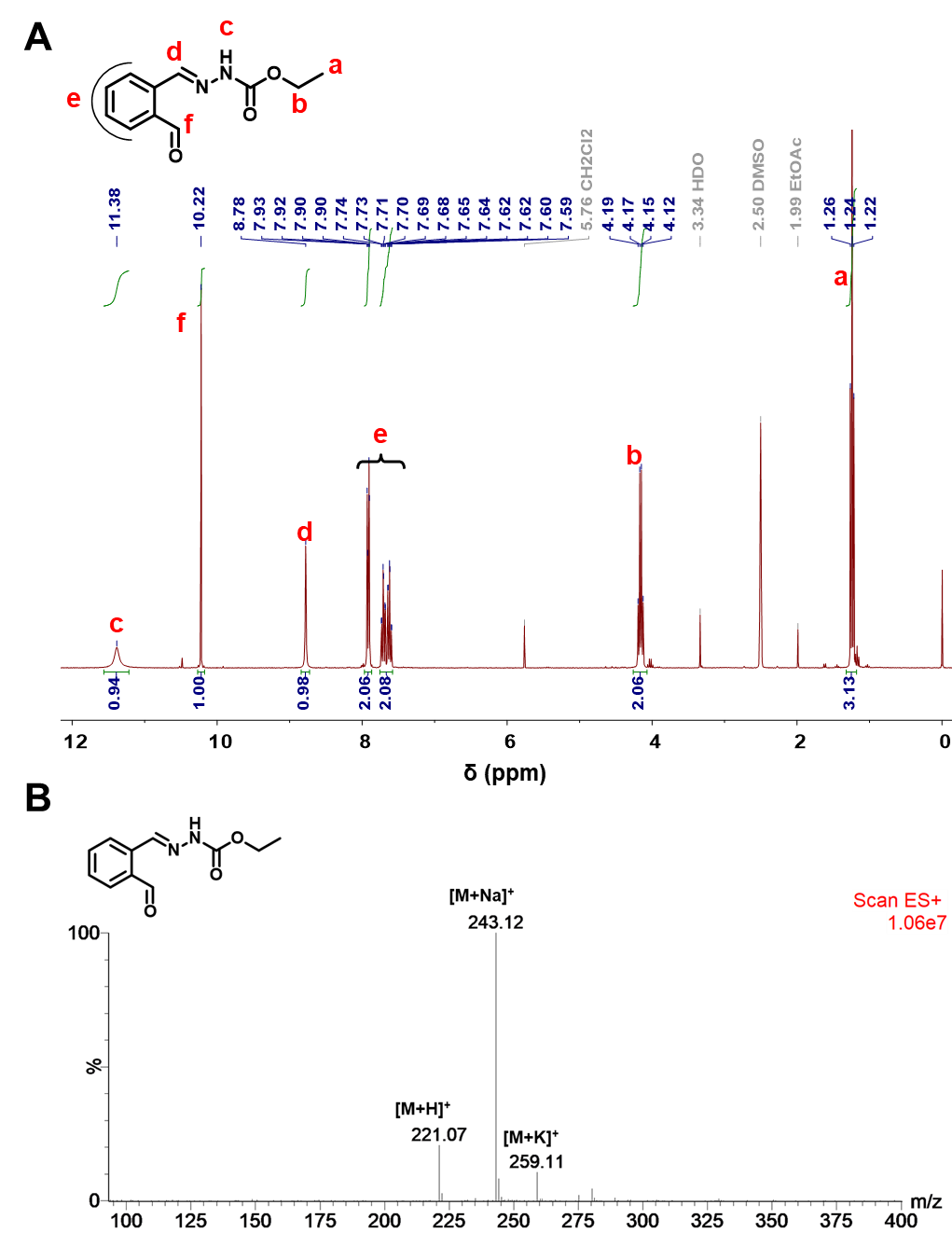


**Figure S23.** (A) ^1^H NMR spectrum of mono-hydrazone in DMSO-*d*_6_. (B) ESI-MS spectrum of mono-hydrazone.


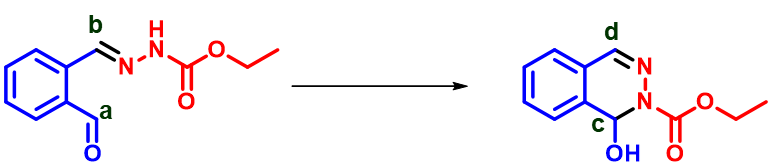


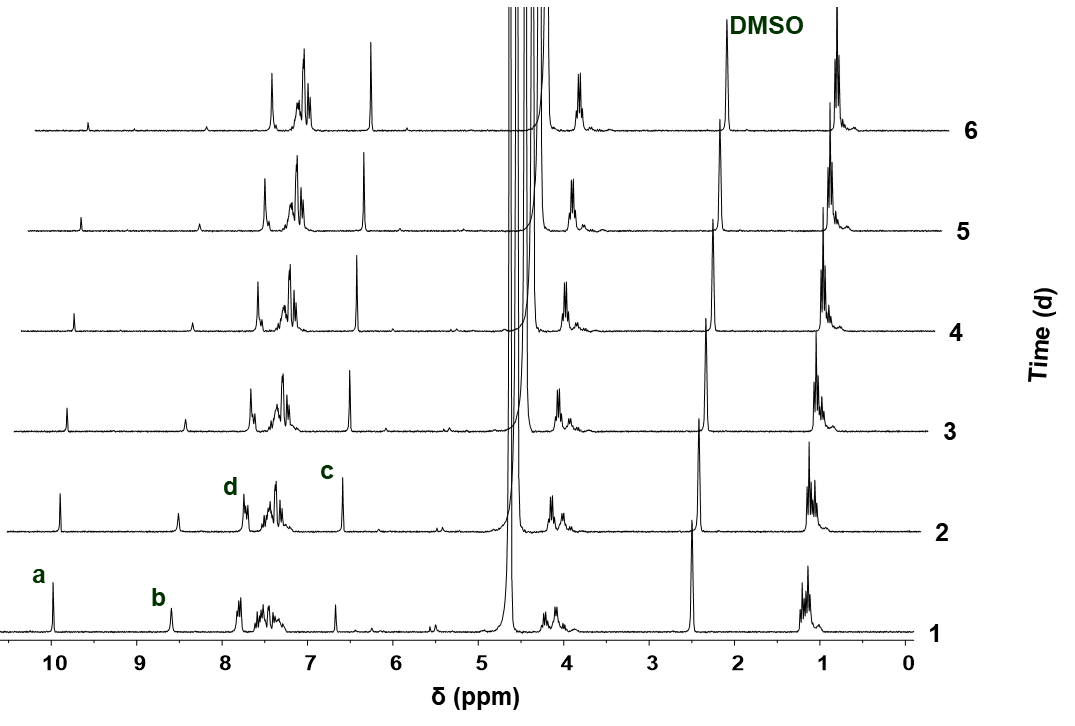


**Figure S24.** Time-dependent ^1^H NMR spectra suggesting the intramolecular cyclization of mono-hydrazone to form 2-(ethoxycarbonyl)-1,2-dihydro-phthalazin-1-ol in D_2_O/DMSO-*d*_6_ (4:1 (v/v)).


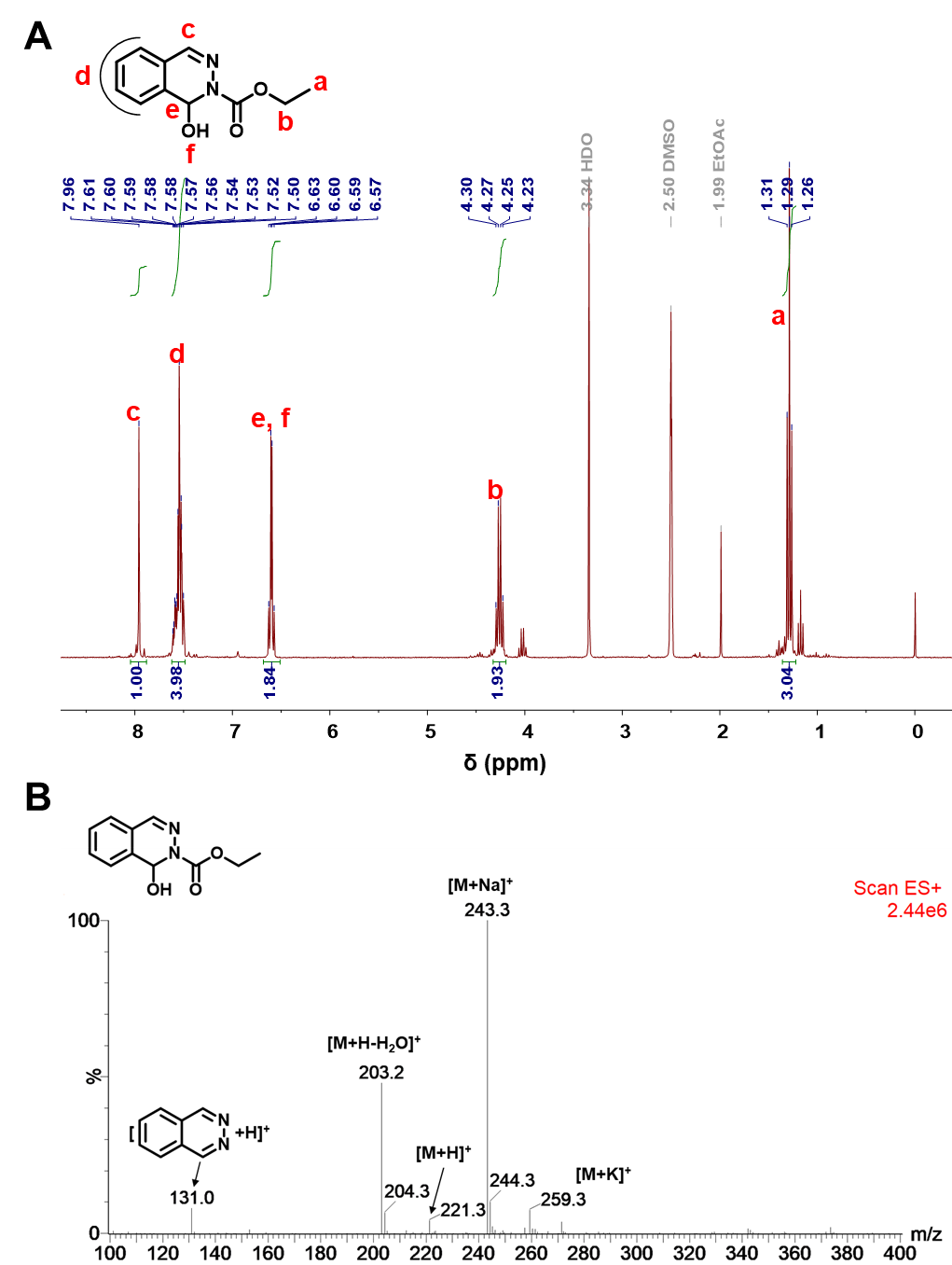


**Figure S25.** (A) ^1^H NMR spectrum of cyclization product in DMSO-*d*_6_. (B) ESI-MS spectrum of cyclization product.


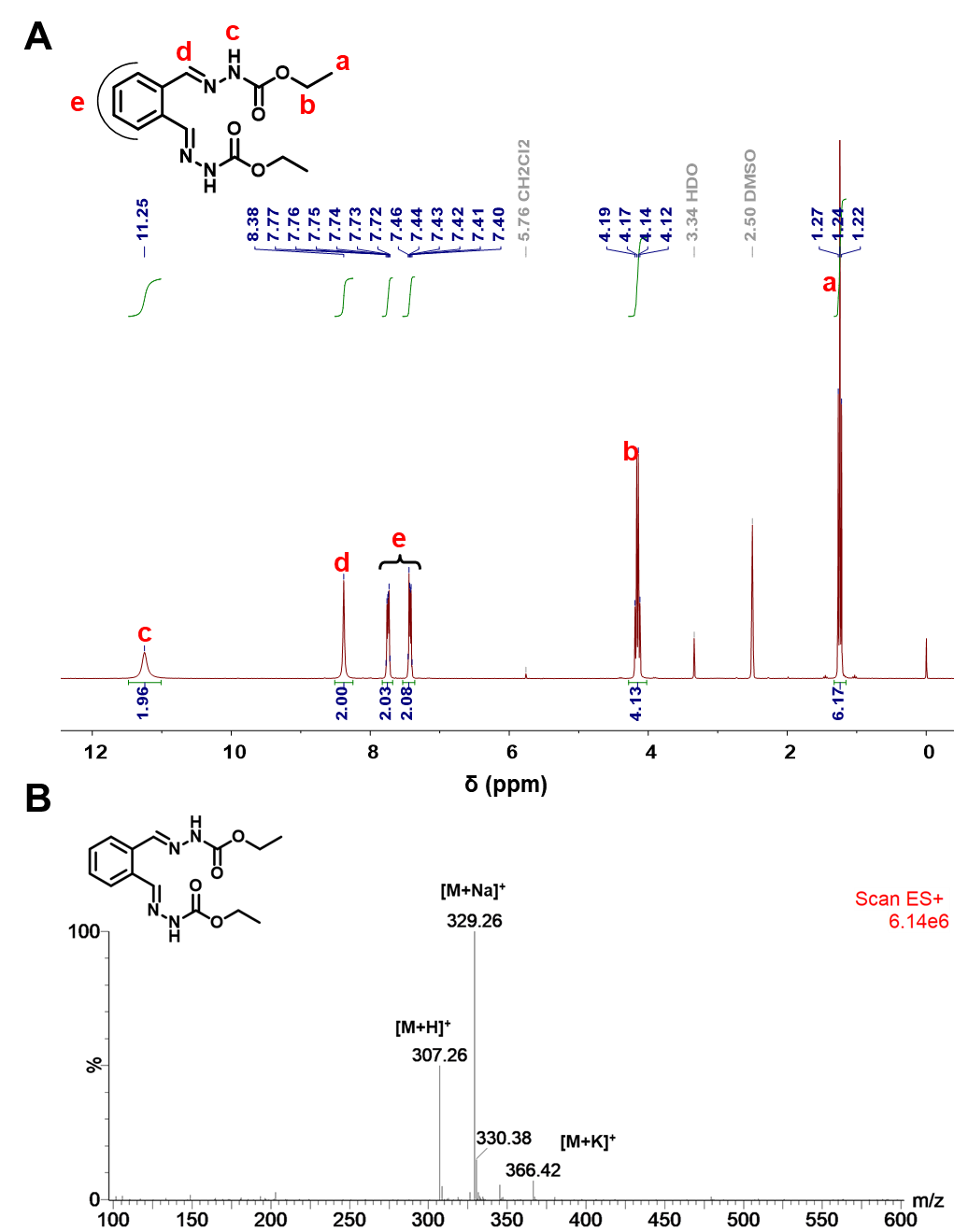


**Figure S26.** (A) ^1^H NMR spectrum of bis-hydrozone in DMSO-*d*_6_. (B) ESI-MS spectrum of bis-hydrozone.


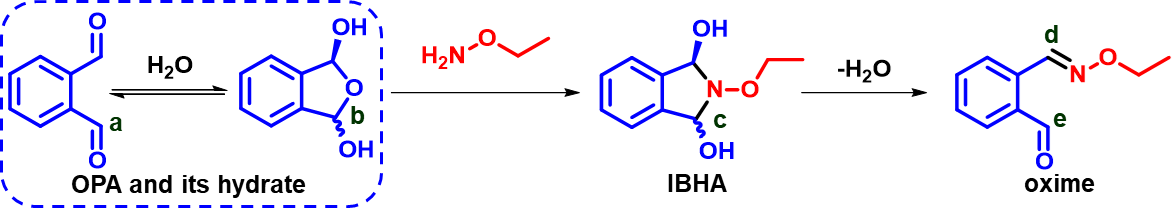


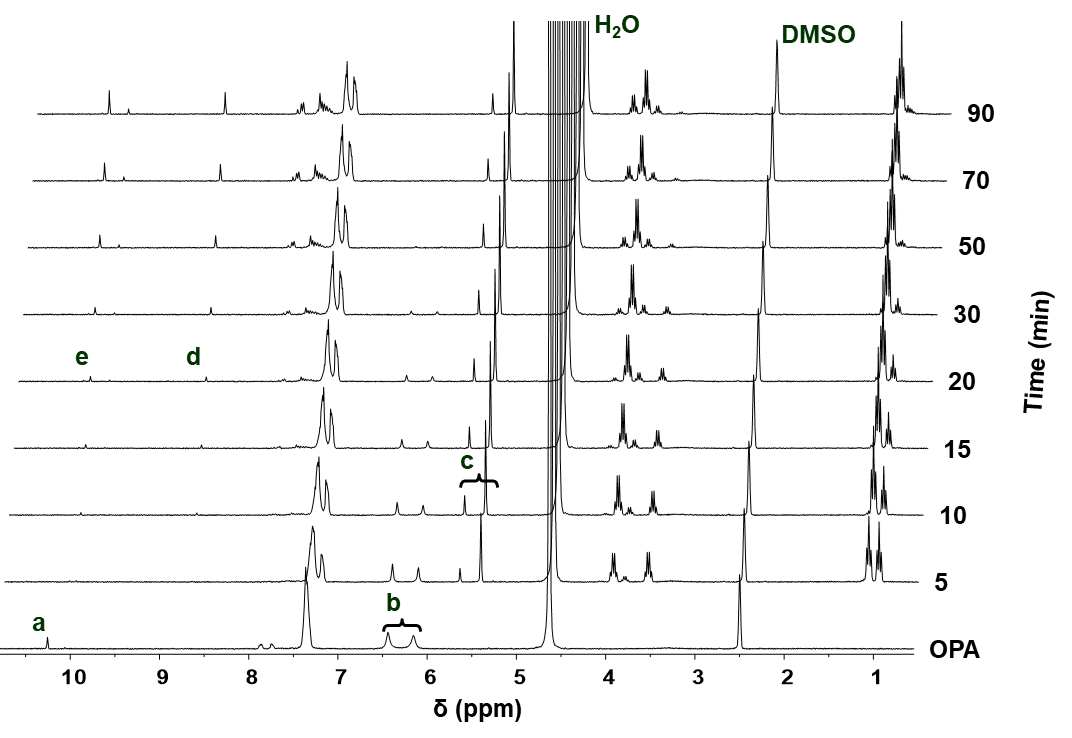


**Figure S27.** Time-dependent ^1^H NMR analysis of the reaction of OPA with ethoxyamine in D_2_O/DMSO-*d*_6_ (4:1 (v/v)).


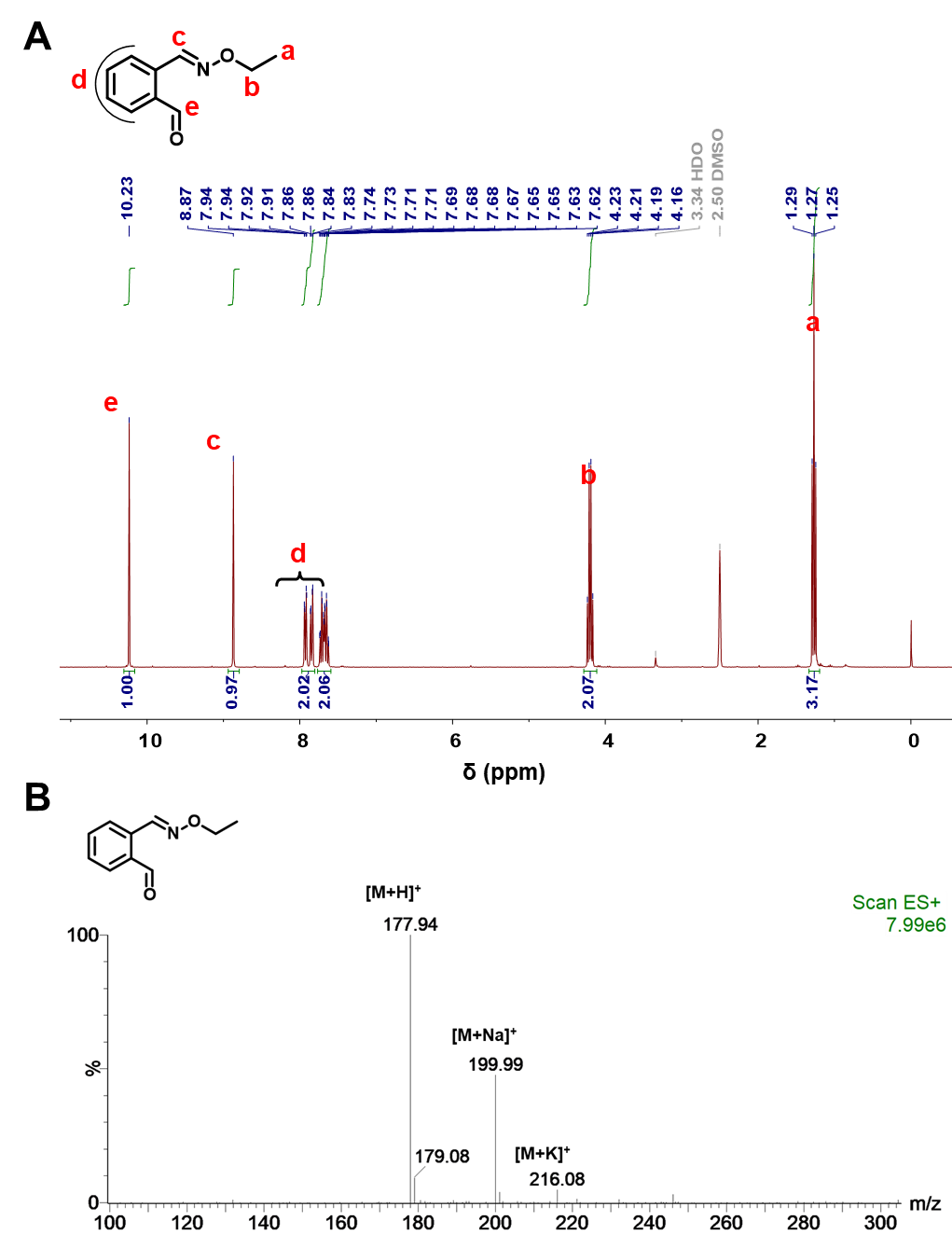


**Figure S28.** (A) ^1^H NMR spectrum of oxime in DMSO-*d*_6_. (B) ESI-MS spectrum of oxime.


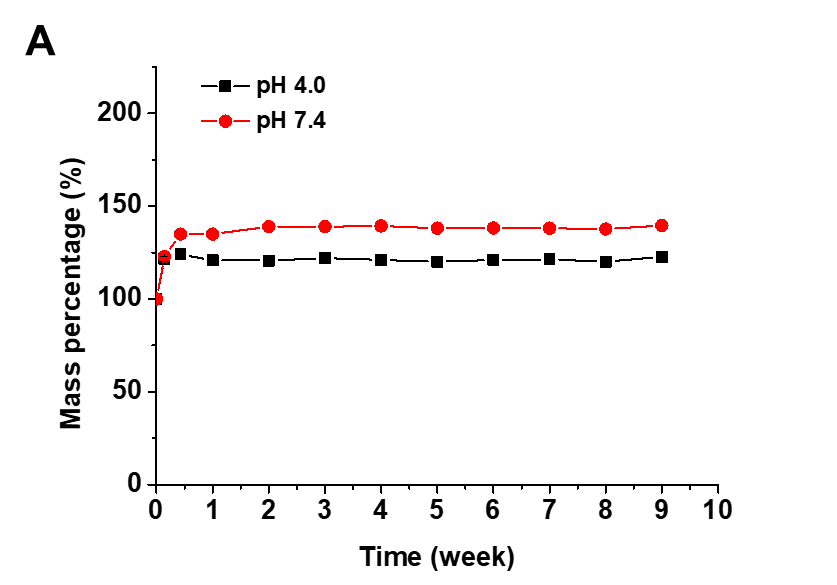


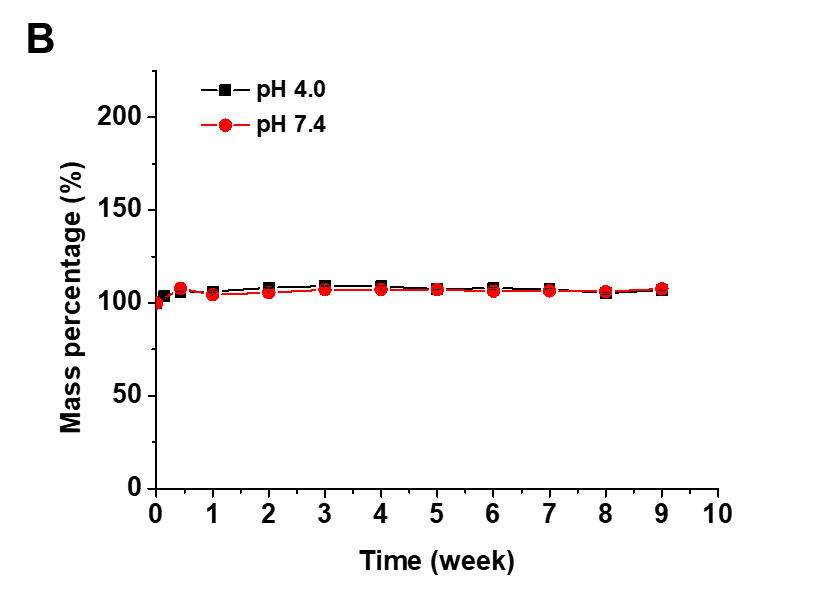


**Figure S29.** Swelling and degradation profiles of (A) 4P-OPA/4P-NH_2_ hydrogels and (B) 4P-OPA/4P-ONH_2_ hydrogels in PBS at different pH values (mean ± SD, n = 3).
